# Highly Effective and Noninvasive Near‐Infrared Eradication of a *Staphylococcus aureus* Biofilm on Implants by a Photoresponsive Coating within 20 Min

**DOI:** 10.1002/advs.201900599

**Published:** 2019-07-19

**Authors:** Mu Li, Liqian Li, Kun Su, Xiangmei Liu, Tianjin Zhang, Yanqin Liang, Doudou Jing, Xianjin Yang, Dong Zheng, Zhenduo Cui, Zhaoyang Li, Shengli Zhu, Kelvin Wai Kwok Yeung, Yufeng Zheng, Xianbao Wang, Shuilin Wu

**Affiliations:** ^1^ Ministry‐of‐Education Key Laboratory for the Green Preparation and Application of Functional Materials Hubei Key Laboratory of Polymer Materials School of Materials Science & Engineering Hubei University Wuhan 430062 China; ^2^ School of Materials Science & Engineering the Key Laboratory of Advanced Ceramics and Machining Technology by the Ministry of Education of China Tianjin University Tianjin 300072 China; ^3^ Department of Orthopaedics Union Hospital Tongji Medical College Huazhong University of Science and Technology Wuhan 430022 China; ^4^ Department of Orthopaedics & Traumatology Li Ka Shing Faculty of Medicine The University of Hong Kong Pokfulam Hong Kong 999077 China; ^5^ State Key Laboratory for Turbulence and Complex System and Department of Materials Science and Engineering College of Engineering Peking University Beijing 100871 China

**Keywords:** antibacterial, biofilms, IR780, MoS_2_, phototherapy

## Abstract

Biofilms have been related to the persistence of infections on medical implants, and these cannot be eradicated because of the resistance of biofilm structures. Therefore, a biocompatible phototherapeutic system is developed composed of MoS_2_, IR780 photosensitizer, and arginine–glycine–aspartic acid–cysteine (RGDC) to safely eradicate biofilms on titanium implants within 20 min. The magnetron‐sputtered MoS_2_ film possesses excellent photothermal properties, and IR780 can produce reactive oxygen species (ROS) with the irradiation of near‐infrared (NIR, λ = 700–1100 nm) light. Consequently, the combination of photothermal therapy (PTT) and photodynamic therapy (PDT), assisted by glutathione oxidation accelerated by NIR light, can provide synergistic and rapid killing of bacteria, i.e., 98.99 ± 0.42% eradication ratio against a *Staphylococcus aureus* biofilm in vivo within 20 min, which is much greater than that of PTT or PDT alone. With the assistance of ROS, the permeability of damaged bacterial membranes increases, and the damaged bacterial membranes become more sensitive to heat, thus accelerating the leakage of proteins from the bacteria. In addition, RGDC can provide excellent biosafety and osteoconductivity, which is confirmed by in vivo animal experiments.

## 1 Introduction

Bacteria tend to colonize and adhere to medical devices, especially in medical implants, to form sessile multicellular communities called as biofilms, leading to persistent and chronic device‐related infectious diseases.^1^, ^2^ For example, *Staphylococcus aureus* (*S. aureus*) accounts for two‐thirds of pathogenic pathogens in orthopedic implant‐related infections, and biofilm formed by *S. aureus* can proliferate and attach to host tissues to cause local purulent infection or even other systemic inflammatory problems.^3^, ^4^, ^5^ Because of the increased antibiotic resistance of bacteria and their efficient mutations to evade the host immune system, implant‐related biofilm infections are difficult to completely eliminate and typically show recurring symptoms even after cycles of traditional antibiotic therapy, and are associated with high mortality rates.^6^, ^7^, ^8^ Therefore, the removal of biofilm‐infected implants is often the only viable remedy, which leads to a second surgery to remove an infected implant. These unsatisfying strategies have prolonged hospitalization periods and increased annual domestic healthcare costs arising from these implant‐related biofilm infections.^9^, ^10^ Considering this concern, great efforts have been made in recent years to develop antimicrobial implant surfaces that rely on the modification of physicochemical properties to interfere with the microbial colonization process. Currently, considering the fact that the initial attachment onto a surface is the major event for biofilm development,^11^, ^12^ the general surface modification strategies for antimicrobial biomaterials involve enhancing the antibacterial activity of the biomaterial itself, i.e., killing bacteria directly through antimicrobial agents in materials, such as loaded drugs, surface charge, and released metallic ions (Ag^+^, Zn^2+^, Cu^2+^, etc.), or resisting bacterial adhesion through electrostatic repulsion and super‐hydrophobicity of surface components. These methods are characterized as “endogenous antimicrobial.” However, endogenous antimicrobial strategies often require much more time to kill bacteria, which inevitably induce the formation of bacterial resistance during repeated actions. In addition to bacterial resistance to organic antibiotics,^13^ inorganic antibacterial agents, even nanosilver, can also be resisted by mutant bacteria during long‐term interactions.^14^ Therefore, it is urgent to develop innovative and creative solutions to eliminate already‐formed biofilms safely without producing bacterial resistance within a short period of time.

Photothermal therapy and photodynamic therapy induced by laser irradiation are attractive emerging therapeutic strategies due to their unique merits, such as nonresistance, few side effects, and low systemic toxicity.^15^, ^16^, ^17^, ^18^, ^19^ Near‐infrared light‐induced hyperthermia for combating bacteria based on photothermal conversion agents is one of the most attractive emerging methods and can destroy bacteria via various thermal effects, such as breakdown of the cell membrane or denaturation of proteins/enzymes.^20^, ^21^ Recent researches have demonstrated that semiconductors and inorganic nanoparticles have the potential to convert light into heat upon irradiation. For example, gold nanoparticles^22^, ^23^ and reduced graphene oxide^24^, ^25^ have been widely used as photothermal conversion agents for combating bacteria in combination with laser light because of its strong light‐absorbing properties. However, effective photothermal antibacterial efficacy only relies on high temperature (e.g., *S. aureus*, a gram‐positive bacteria, exhibits great heat resistance),^26^ and long‐time irradiation combined with a high‐power density of light would result in tissue burns, which is a challenge associated with photothermal therapy (PTT). Moreover, reactive oxygen species (ROS) has been reported to induce bacteria death through the initial oxidative lesions effect on cell membrane and wall.^27^ However, if the imbalance between ROS generation and detoxification leads to high levels of ROS, it could generate oxidative stress on cells greatly, which may result in cellular constituents damage (such as proteins, DNA, and lipids) and lead to apoptosis, or even cause the promotion of cancer mutations.^28^, ^29^

**Figure 1:**
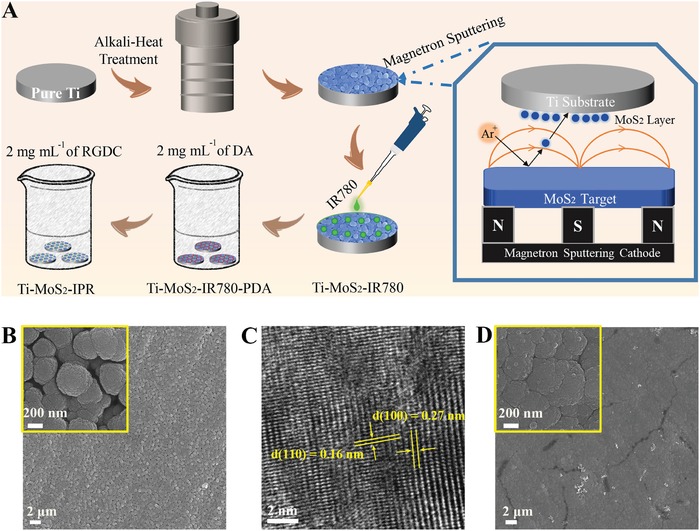
A) The drawing of fabrication process of the MoS_2_–IR780–PDA–RGDC coating on Ti plates. B) FE‐SEM images of magnetron‐sputtered MoS_2_ coating on Ti plates. C) HRTEM images of MoS_2_ film separated from Ti–MoS_2_. D) FE‐SEM images of Ti–MoS_2_–IR780–PDA–RGDC (Ti–MoS_2_–IPR) plates.

Multiple synergetic antibacterial modalities are considered promising approaches to induce potent synergistic effect and decrease the side effect of single modality. In this study, we proposed a synergistic exogenous antimicrobial system composed of MoS_2_, IR780, and arginine–glycine–aspartic acid–cysteine (RGDC), in which MoS_2_ is a biocompatible prototypical transition‐metal dichalcogenide that exhibits high photothermal conversion efficiency,^30^, ^31^, ^32^, ^33^ while IR780 is a photosensitizer that can transfer the energy of near‐infrared (NIR) light to dissolved oxygen (^3^O_2_) to generate singlet oxygen (^1^O_2_).^34^, ^35^ By electrostatic binding forces, the IR780 photosensitizer with positive charge can adsorb into the negatively charged MoS_2_ layer. RGDC is natural bioactive material that can promote osteoconductivity,^36^, ^37^ and can also be grafted onto Ti plates through a reaction with polydopamine (PDA) that can be used as the reaction points for grafting modification.^38^, ^39^ The process for material preparation is schematically shown in **Figure** 1A. We hypothesize that under NIR irradiation, moderate hyperthermia and ^1^O_2_ from this system can produce much higher synergistic phototherapeutic efficacy with excellent biosafety for biofilm‐infected implants compared with PTT or photodynamic therapy (PDT) alone.

## 2 Results and Discussion

### 2.1 The Characteristic of Morphology and Structure

The field emission scanning electron microscopy (FE‐SEM) was employed to observe the surface morphology of the samples. A porous network was formed on the Ti plates after alkali‐heat reaction (Figure S1A, Supporting Information), and the major elements included Ti and O (Figure S1B, Supporting Information). As shown in Figure 1B, the magnetron‐sputtered MoS_2_ was distributed on the Ti plates uniformly and compactly, which was indicated by elemental mapping through energy disperse spectroscopy (EDS), as shown in Figure S2 of the Supporting Information (Mo in green, S in purple). EDS image showed that the atomic ratio of Mo/S was ≈13.51: 25.06 in the coating (Figure S3, Supporting Information), which was consistent with the composition of MoS_2_. As shown in Figure 1C, the high‐resolution transmission electron microscopy (HRTEM) image showed the periodic honeycomb arrangement of atoms with interplanar spacings of ≈0.27 and ≈0.16 nm for (100) and (110) lattice orientations, respectively.^40^, ^41^ Furthermore, X‐ray diffraction (XRD) patterns further confirmed that the MoS_2_ coating was prepared successfully (Figure S4, Supporting Information), and the detected peaks located at 33.8° and 60.0° could be attributed to the (100) and (110) planes in MoS_2_, respectively.^42^, ^43^ The cross‐section image showed that the thickness of this film was ≈1.26 µm (Figure S5, Supporting Information). The subsequent electrostatic bonding of IR780 and covalent immobilization of the RGDC peptide through PDA filled the gaps between the MoS_2_ spheres to form a relatively smooth surface morphology (Figure 1D). Elemental mapping images of the prepared sample Ti–MoS_2_–IR780–PDA–RGDC (Ti–MoS_2_–IPR) showed a homogeneous distribution of elements (Figure S6A, Supporting Information), the appearance of Cl and I confirmed the successful grafting of IR780 with a low‐element content (Figure S6B, Supporting Information), and the chemical formula of the IR780 was shown in Figure S6C, Supporting Information.

**Figure 2:**
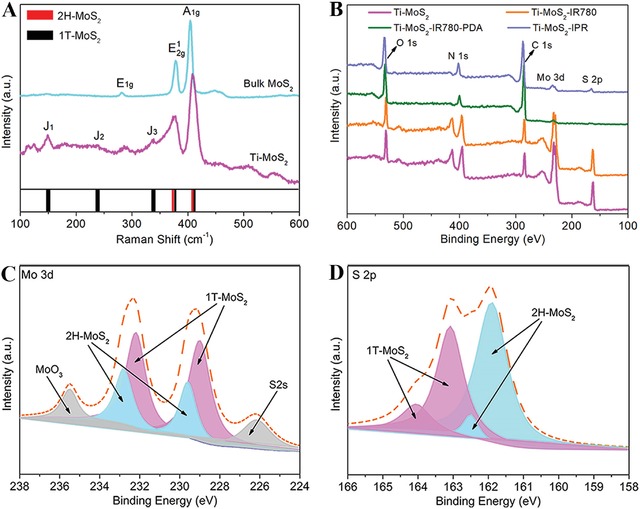
A) Raman spectra of bulk MoS_2_ and Ti–MoS_2_. B) XPS survey spectra of Ti–MoS_2_, Ti–MoS_2_–IR780, Ti–MoS_2_–IR780–PDA, and Ti–MoS_2_–IPR. XPS narrow scan of C) Mo 3d and D) S 2p obtained from Ti–MoS_2_ plates.

The phase identity of the as‐synthesized magnetron‐sputtered MoS_2_ coating was further confirmed using Raman spectroscopy in the region of 100–600 cm^−1^, which was shown in **Figure** 2A. The Raman spectra showed that the magnetron‐sputtered MoS_2_ coating on Ti had typical 2H‐MoS_2_ and 1T‐MoS_2_ bands. The characteristic Raman shifts at 375 and 407 cm^−1^ expected for the E^1^_2g_ and A_1g_ of 2H‐MoS_2_ were clearly observed. Compared with bulk MoS_2_, the emergence of new Raman shifted at 148, 237, and 337 cm^−1^ for *J*_1_, *J*_2_, and *J*_3_, respectively, was associated with the phonon modes of 1T‐MoS_2_.^44^, ^45^ As shown in Figure 2B, the X‐ray photoelectron spectroscopy (XPS) spectra showed the significant signal peaks of Mo 3d and S 2p, which were attributed to the magnetron‐sputtered MoS_2_ coating. Obviously, the peak intensity in Mo 3d and S 2p decreased significantly after PDA modification. After immobilization of the RGDC peptide, the intensity of the S 2p peak increased compared to Ti–MoS_2_–IR780–PDA, indicating that the sample was successfully modified with RGDC. Furthermore, the XPS narrow spectra obtained from the Ti–MoS_2_ sample showed that the binding energies of the Mo 3d peaks could be deconvoluted into four subpeaks at 232.8, 229.6, 232.2, and 229.0 eV, further confirming the 2H‐MoS_2_ and 1T‐MoS_2_ phases (Figure 2C).^46^, ^47^ The corresponding XPS narrow spectra of S 2p identified at 161.9, 162.5, 163.1, and 164.0 eV were shown in Figure 2D, and were assigned to 2H‐MoS_2_ and 1T‐MoS_2_. In addition, the C 1s peaks (Figure S7A, Supporting Information) located at 284.1, 284.6, 285.4, and 286.5 eV were corresponded to C=C, C—C, C—N, and C—Cl respectively, indicating the electrostatic combination of MoS_2_ and IR780. The appearance of the C—O peak obtained from Ti–MoS_2_–IR780–PDA at 286.3 eV in Figure S7B (Supporting Information) indicated the PDA modification. The C=O peak appeared at 288.0 eV in Figure S7C (Supporting Information) was attributed to the amide bond of RGDC, further confirming the successful grafting of RGDC.

In addition to XPS, the layer‐by‐layer surface modification process can also be indicated by the variation in the contact angle at different stages. Figure S8 (Supporting Information) showed that the alkali‐heat‐treated Ti plates had a contact angle of 14.0° ± 0.5°. The subsequent‐magnetron sputtered MoS_2_ coating increased the contact angle to 81.6° ± 0.62°, which was likely due to the hydrophobicity of MoS_2_.^48^ The subsequent electrostatic bonding of IR780 further increased the contact value to 89.0° ± 2.5°, attributing to the hydrophobicity of IR780.^35^ Because of the hydrophilicity of PDA and RGDC,^37^, ^39^ the subsequent grafting of PDA and RGDC induced the contact angles to 50.4° ± 0.12 and 29.1° ± 0.88°, respectively. In addition, Figure S9 (Supporting Information) showed the binding force analysis of hybrid coating and the Ti substrate. There existed distinct scratches clearly on the Ti–MoS_2_ and Ti–MoS_2_–IPR samples (Figure S9A,B, Supporting Information). According to Figure S9C,D (Supporting Information), the average lateral force in these two samples showed nearly identical force (about 2.3 mN), indicating strong bonding between the substrate and the coatings.

### 2.2 In Vitro Photothermal Effects and Reactive Oxygen Species Detection

**Figure 3:**
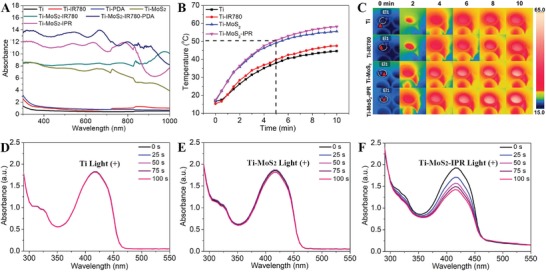
A) UV–Vis–NIR absorbance spectra of Ti, Ti–IR780, Ti–PDA, Ti–MoS_2_, Ti–MoS_2_–IR780, Ti–MoS_2_–IR780–PDA, and Ti–MoS_2_–IPR. B) Heating experiments of Ti, Ti–IR780, Ti–MoS_2_, and Ti–MoS_2_–IPR in 100 µL of PBS aqueous solution under NIR light irradiation (10 min, 0.5 W cm^−2^). C) The corresponding real‐time infrared thermal images of different samples immersed into PBS aqueous solution under continuous light irradiation. The decay of DPBF for the detection of ^1^O_2_ in D) Ti, E) Ti–MoS_2_, and F) Ti–MoS_2_–IPR with light irradiation (100 s, 0.5 W cm^−2^). The experiments are performed in triplicate and independently.

As shown in **Figure** 3A, both Ti–MoS_2_ and Ti–MoS_2_–IPR exhibited clearly enhanced absorption in the NIR region compared to pure Ti. Compared to Ti–MoS_2_, the absorption of Ti–MoS_2_–IPR was increased, which was likely attributed to the IR780 and PDA NIR absorption.^20^, ^35^ Heating temperature curves of samples were showed in Figure 3B, with the samples immersed in 100 µL of phosphate buffered saline (PBS) and irradiated by NIR light for 10 min (0.5 W cm^−2^). After 5 min of irradiation, the surface temperatures of Ti–MoS_2_ and Ti–MoS_2_–IPR increased to 49.2 and 50.6 °C, respectively. By contrast, the temperatures of Ti and Ti–IR780 only increased to 38.0 and 39.9 °C, respectively, with the same light irradiation conditions, indicating that the main source of photothermy is attributed to MoS_2_ instead of photosensitizer of the IR780 photosensitizer or Ti. As shown in Figure 3C, the corresponding photothermal images of the samples demonstrated the significant photothermal conversion efficiency of Ti–MoS_2_–IPR. The laser on–off cycles in Figure S10 (Supporting Information) disclosed that repeated irradiation did not influence the photothermal properties of Ti–MoS_2_–IPR, indicating the photostability of the prepared Ti–MoS_2_–IPR system.

1,3‐Diphenylisobenzofuran (DPBF), used as ^1^O_2_‐trapping agent, reacts quickly with ^1^O_2_ to form a product with a decreased absorption intensity centered around 410 nm.^49^, ^50^ For the Ti and Ti–MoS_2_ groups, the UV absorption peaks of DPBF solution showed negligible changes after irradiation for 100 s under NIR light (0.5 W cm^−2^) (Figure 3D,E) or after treatment in the dark (Figure S11A,B, Supporting Information) for 100 s. By contrast, the absorption intensity decreased gradually for Ti–MoS_2_–IPR sample with light irradiation (0.5 W cm^−2^) (Figure 3F), indicating the significant generation of ^1^O_2_. The absorbance of DPBF in the Ti–MoS_2_–IPR group exhibited a negligible change in the absence of light (Figure S11C, Supporting Information), suggesting the light‐dependent features of the generation of ^1^O_2_. A similar negligible change in the absorption intensity of the DPBF dye at 50 °C was shown in Figure S11D (Supporting Information), indicating that the dye was very thermally stable. Therefore, these results demonstrate that both photothermal and photodynamic effects are produced under irradiation by a single light source.

### 2.3 In Vitro Antibacterial Activity

**Figure 4:**
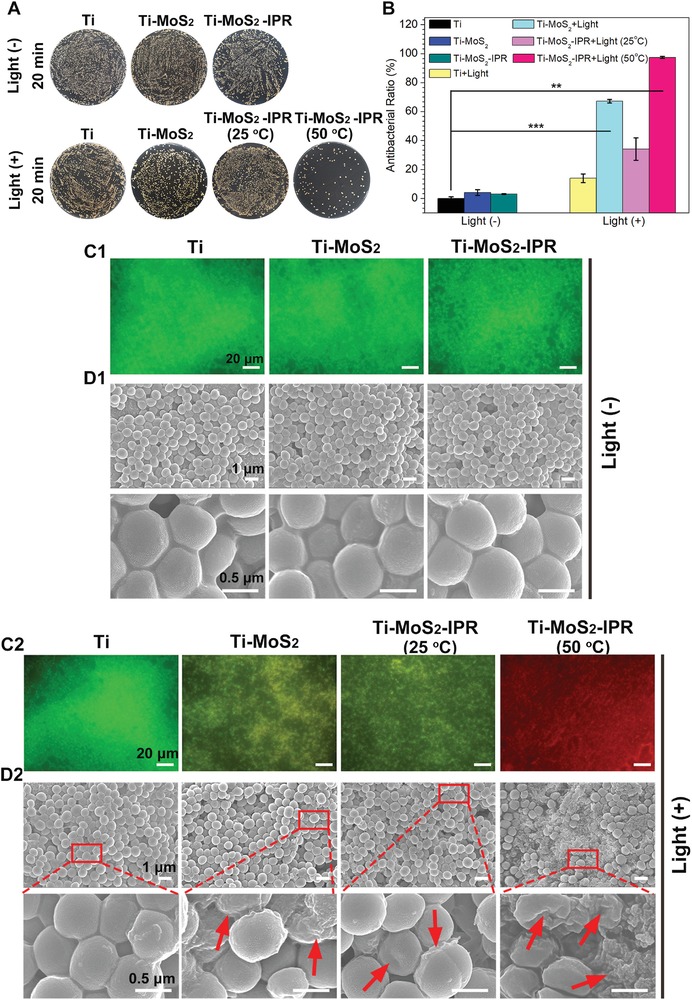
A) Spread plate results of *S. aureus* biofilm eradication in different samples after treatment in the dark or irradiation with NIR light for 20 min. B) The corresponding antibacterial ratio of Ti, Ti–MoS_2_, Ti–MoS_2_–IPR, Ti+Light, Ti–MoS_2_+Light, Ti–MoS_2_–IPR+Light (25 °C), and Ti–MoS_2_–IPR+Light (50 °C) groups, student *t*‐test. C1,C2) Fluorescence images of biofilms treated with or without light irradiation, in which dead cells stained in red and live cells stained in green. D1,D2) FE‐SEM images of the morphology of biofilms treated with or without light irradiation. Error bars indicate means ± standard deviations: **p < 0.01, ***p < 0.001.

Spread plate method was employed to determine the antibiofilm efficiency against the biofilm. The Ti, Ti–MoS_2_, and Ti–MoS_2_–IPR groups showed nearly identical bacterial colonies after culture in the dark for 20 min (**Figure** 4A), and compared to pure Ti, the corresponding antibacterial ratios were 4.04 ± 1.41% for Ti–MoS_2_ and 2.97 ± 0.22% for Ti–MoS_2_–IPR (Figure 4B). These results indicate that the samples have a negligible effect on the biofilm in the absence of light irradiation. In comparison, after irradiated by NIR light for 20 min, bacterial colonies on the Ti–MoS_2_+Light, Ti–MoS_2_–IPR+Light (25 °C), and Ti–MoS_2_–IPR+Light (50 °C) groups exhibited varying degrees of decline, and the antibacterial efficacies were 67.19 ± 0.85%, 34.05 ± 5.4%, and 97.41 ± 0.46% against the *S. aureus* biofilm, respectively (Figure 4B). However, the bacterial colonies on the Ti+Light group showed a negligible change, suggesting that the effect of NIR light on the survival of the bacteria is negligible. These results also suggest that the individual PTT on the *S. aureus* biofilm is more effective than PDT alone, as demonstrated by the spread plate results of the Ti–MoS_2_+Light and Ti–MoS_2_–IPR+Light (25 °C) groups. Therefore, a single‐modal antibacterial process is proven to be very difficult to completely and efficiently eradicate the already‐formed biofilm. The combination of PTT and PDT could exhibit much higher antibacterial efficacy on *S. aureus* biofilms than PTT or PDT alone.

Live/dead (green/red) staining assays were employed to qualitatively evaluate the synergistic antibacterial efficiency of samples, and the corresponding fluorescent photographs of the *S. aureus* biofilm were shown in Figure 4C1,C2. Without light irradiation, the surface on the Ti, Ti–MoS_2_, and Ti–MoS_2_–IPR groups were completely stained with green, showing that bacteria colonized the surface of samples and formed sessile multicellular communities, indicating that all samples are suitable for bacterial growth when cultured in the dark. After illumination with 808 nm light for 20 min, the Ti+Light group showed similar fluorescence compared with the groups cultured in the dark, suggesting the whole alive biofilm morphology. By contrast, some yellow fluorescence (overlay of green and red) appeared on the Ti–MoS_2_+Light and Ti–MoS_2_–IPR+Light (25 °C) groups. By contrast, no green fluorescent spots were observed for the Ti–MoS_2_–IPR+Light (50 °C) group, indicating the best efficacy of eradiating the bacterial biofilm, which is consistent with spread plates results.

The morphologies and membrane integrity of the adherent *S. aureus* biofilm were examined by FE‐SEM (Figure 4D1,D2). After culturing in the dark for 20 min, the *S. aureus* biofilms showed a compact morphology with a smooth and integrated surface on all three kinds of samples, demonstrating little toxicity against bacteria. In comparison, after irradiated by NIR light for 20 min, the *S. aureus* cells showed varying degrees of deformation, as indicated by membrane damage of the bacteria cultured in the Ti+Light, Ti–MoS_2_+Light, and Ti–MoS_2_–IPR+Light (25 °C) groups, and more serious membrane shrinkage or even cracking was observed for bacteria cultured in the Ti–MoS_2_–IPR+Light (50 °C) group (marked by red arrows in Figure 4D1,4D2). In conclusion, these results refer that synergetic action of photothermy and ^1^O_2_ are responsible for the effective antibiofilm activity within a short period of time.

**Figure 5:**
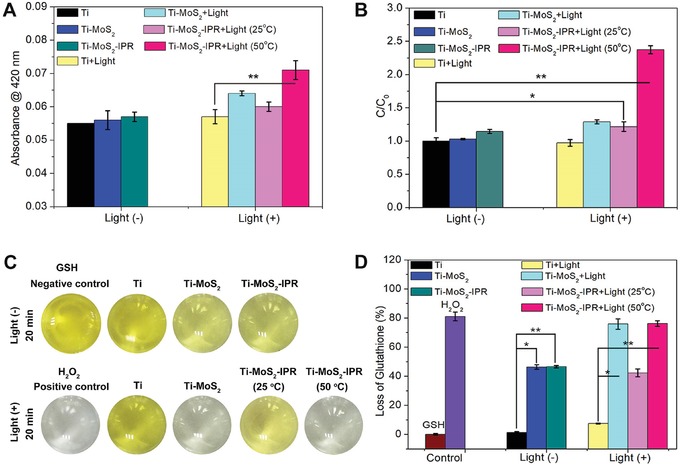
A) Results of the ONPG hydrolysis assay for investigating the change of bacterial cell membrane permeability, student *t*‐test. B) A histogram of the relative protein leakage concentration for the bacteria treated or not treated with 808 nm light irradiation, student *t*‐test. C) Images of the color changes in GSH solutions after incubation with samples for 20 min. D) The corresponding loss of GSH in different samples, student *t*‐test. *p < 0.05, **p < 0.01.

The antibacterial mechanism of multiple synergetic antibacterial modalities was confirmed by *ortho*‐nitrophenyl‐β‐galactoside (ONPG) hydrolysis assays, which can be used to evaluate the change in cell membrane permeability.^51^, ^52^ As shown in **Figure** 5A, hydrolysis of ONPG in the Ti group exhibited a negligible change regardless of light, suggesting that the Ti group exhibited negligible effect on the bacteria membrane. By contrast, the Ti–MoS_2_+Light and Ti–MoS_2_–IPR+Light (25 °C) groups exhibited increased hydrolysis of ONPG compared to Ti group, which indicated that both photothermy and ^1^O_2_ can improve bacterial membrane permeability. The membrane permeability improves significantly in the Ti–MoS_2_–IPR+Light (50 °C) group after combining PTT and PDT, suggesting that oxidative lesions induced by a small quantity of ROS can greatly assist photothermal therapy. This synergistic effect on bacteria was further confirmed by the protein leakage analysis in Figure 5B, showing the same tendency as the hydrolysis of ONPG.

Glutathione (GSH), a tripeptide consisting of glutamate, glycine, and cysteine residues, is the primary endogenous antioxidant in cells, and it can also act as a cell oxidative stress indicator.^53^, ^54^ To investigate oxidative stress induced by our samples, Ellman's assay was employed to measure the GSH oxidation. Figure 5C showed the images of the color change in GSH solutions after incubation with different samples. The Ti group showed negligible color changes regardless of light irradiation, suggesting that the Ti group had no oxidation effect. For the Ti–MoS_2_ and Ti–MoS_2_–IPR groups, the yellow color faded slightly after treatment in the dark for 20 min, and the corresponding loss of GSH was 46.33 ± 1.05% and 46.51 ± 0.60%, respectively (Figure 5D). The catalysis of MoS_2_ has been reported for the oxidation of organic thiols (R‐SH) to yield disulfides (R‐S‐S‐R).^27^ Therefore, the loss of GSH in the Ti–MoS_2_ and Ti–MoS_2_–IPR groups in the dark was attributed to the oxidative lesions of MoS_2_. Moreover, the color in the Ti–MoS_2_+Light and Ti–MoS_2_–IPR+Light (50 °C) groups nearly completely subsided. The corresponding loss of GSH was 75.86 ± 2.52% and 76.22 ± 1.33%, respectively (Figure 5D). By contrast, the loss of GSH in the Ti–MoS_2_–IPR+Light (25 °C) group was 42.26 ± 1.89%. Therefore, the results demonstrate the MoS_2_‐related coating for the catalytic of GSH oxidation, which explains the rapid bacterial death observed under light irradiation.

**Scheme 1:**
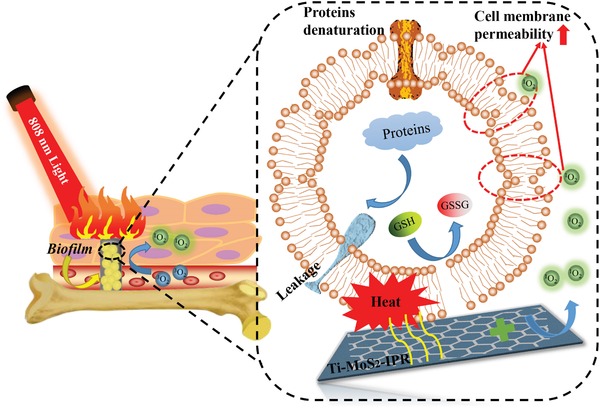
The illustration of *S. aureus* biofilm eradication process with NIR light irradiation.

Herein, a mechanism based on exogenous ROS‐enhanced PTT for eradicating already‐formed biofilms is shown in **Scheme** 1. Following irradiation by NIR light, a small quantity of ROS interacts with bacteria to induce initial oxidative lesions on the cell membranes and walls or even compromise membrane integrity, causing bacteria to be more vulnerable to heat. With the combination of PTT, heat can destroy bacteria more easily, which could minimize the side effects of a single‐modal antibacterial process based on high temperatures. In addition, hyperthermia induced by NIR light can accelerate GSH oxidation significantly, which breaks down the bacterial antioxidant defense system, thus contributing to the increase in antibacterial efficiency against *S. aureus* biofilms with light irradiation.

### 2.4 In Vitro Cytocompatibility

**Figure 6:**
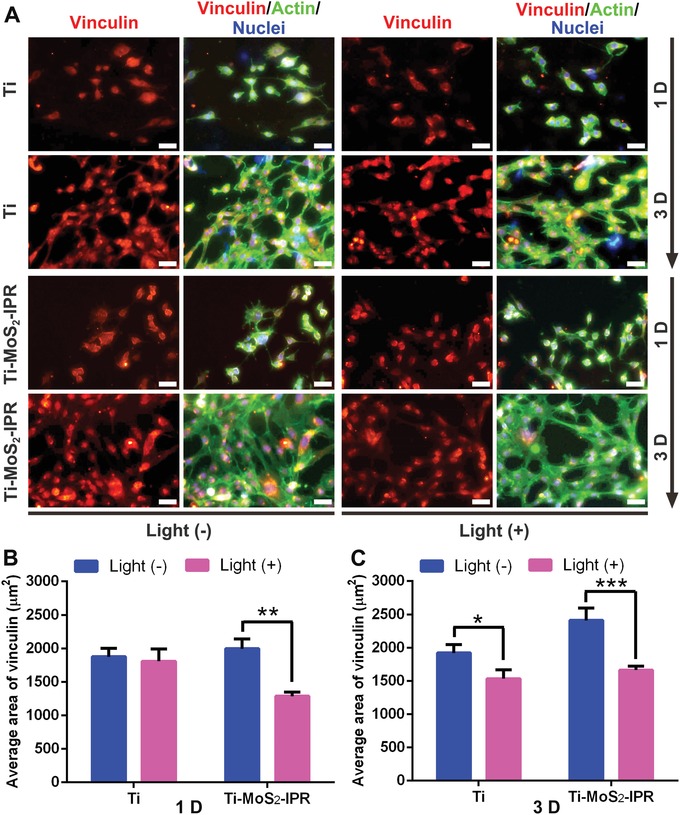
A) Micrographs of time‐dependent immnuofluorescence staining against vinculin (red), actin (green), and nuclei (blue) of fibroblast on different substrates irradiated with or without 808 nm light (scale bars: 50 µm). B,C) Quantitative analysis of vinculin at 1 and 3 days, two‐way ANOVA with Sidak's multiple comparisons test. *p < 0.05, **p < 0.01, and ***p < 0.001.

Cell morphology and spreading activity for sensing and responding to the surrounding microenvironment were observed by FE‐SEM. Figure S12 (Supporting Information) showed that NIH‐3T3 cells (Tongji Hospital, Wuhan) adhered to Ti, Ti–MoS_2_, and Ti–MoS_2_–IPR after being cultured in the dark for 3 days. However, the cells exhibited improved spreading on the surface of Ti–MoS_2_–IPR, and most of the cells had a fair amount of filopodia and lamellipodia compared with cells on the Ti group, which had a shape with smooth margins. Moreover, the cells adhered to Ti–MoS_2_ also spread poorly, with a shrinking and dendric shape. To determine the influence of NIR light on cell proliferation, the methyl thiazolyl tetrazolium (MTT) assay was performed with or without 808 nm light irradiation. Figure S13 (Supporting Information) showed that throughout the incubation periods of 3 days without initial light irradiation, both Ti–MoS_2_ and Ti–MoS_2_–IPR groups stimulated the proliferation of fibroblasts compared to pure Ti, and the corresponding cell viability increased to ≈173.29% and 207.51%, respectively. By contrast, the cell viability of the light‐irradiated Ti group was ≈98.65% compared to unexposed pure Ti group, indicating that the light had negligible negative effect on the survival of healthy cells. As for Ti–MoS_2_ and Ti–MoS_2_–IPR groups, the corresponding cell viability of the light‐irradiated groups decreased mildly compared to the nonlight groups, suggesting that the photothermal (Figure 3B) and photodynamic (Figure 3F) effects under light irradiation damaged cells to a certain extent compared to the groups treated in the dark. And the relative higher cell viability on Ti–MoS_2_–IPR group is probably due to the modification of the RGDC. It is because the effects of light irradiation are temporary, and cell damage can be restored over time due to the biocompatibility of the samples. Vinculin is a critical component of focal adhesions, which exhibits a crucial role in cell sensing and adhesion.^55^, ^56^ As shown in **Figure** 6A, we observed that the cells grew on the Ti and Ti–MoS_2_–IPR substrates after being cultured for 1 and 3 days, with different extents of immunofluorescence. For the Ti group, the average area of vinculin (1875.82 µm^2^) changed negligibly after light irradiation (Figure 6B). By contrast, cells adhered to the Ti–MoS_2_–IPR+Light group developed a smaller spherical morphology with disappearance of the spindle‐like podosome, and the corresponding average area of vinculin shrunk from 1994.47 to 1287.12 µm^2^. After incubation for 3 days, the cells proliferated on the samples, and the cells grown on Ti–MoS_2_–IPR developed more pronounced actin fibers and an increased average area of vinculin. Surprisingly, the average area of vinculin in the Ti–MoS_2_–IPR+Light group increased to 1664.68 µm^2^, and the cells contained large number of filopodia and lamellipodia (Figure 6C), indicating that short periods of light exposure can affect cell growth to a slightly extent. In conclusion, the cytotoxicity of both photothermy and ^1^O_2_ shows light and time‐dependent features, so the cells would become normal and spread evenly because of the good biocompatibility of the samples when the culturing time increases without light irradiation.

### 2.5 In Vivo Biofilm Eradication

**Figure 7:**
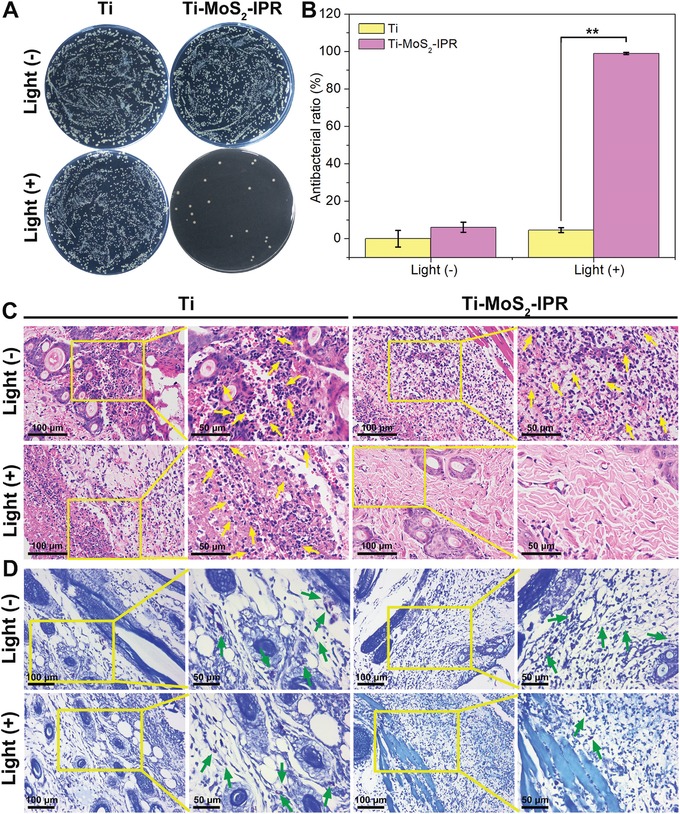
A) Spread plate results of the *S. aureus* biofilm implant after treatment in the dark or after irradiation with NIR light for 20 min in vivo. B) The corresponding in vivo antibacterial efficiency of implants, two‐way ANOVA with Sidak's multiple comparisons test. C,D) H&E and Giemsa staining images show the degree of infection in rats, in which neutrophils marked by yellow arrows and the bacteria marked by green arrows. **p < 0.01.

A subcutaneous infection model using *S. aureus* biofilm‐infected rats was established to evaluate the antibiofilm activity of samples in vivo with the synergetic antibacterial strategy of exogenous ROS‐enhanced PTT. According to the abovementioned results in vitro, pure Ti and Ti–MoS_2_–IPR were used as the control and experimental groups, respectively, for the in vivo animal studies. **Figure** 7 showed the in vivo spread plate results and their corresponding histological analysis of subcutaneous wounds around implants after culture for three days. As shown in Figure S14 (Supporting Information), the wounds treated with the Ti group showed severe bacterial infection with ichor after 20 min light irradiation or after treatment in the dark, indicating that the Ti plates had no effect on the biofilms. The infection condition on the Ti–MoS_2_–IPR group was also observed after treatment in the dark for 20 min. By contrast, no obvious abscess was found in the wounds implanted with the sample from the Ti–MoS_2_–IPR+Light group, indicating that the infection was suppressed and relieved. Furthermore, the residual amount of the biofilm in vivo was investigated by the spread plate method. Figure 7A showed that there were few bacterial colonies on the Ti–MoS_2_–IPR+Light group, and the calculated efficiency of resisting the *S. aureus* biofilm reached 98.99 ± 0.42% in vivo compared to pure Ti (Figure 7B).

The amount of immune cells, such as neutrophils, indicates the bacterial infection level in tissues.^57^ Figure 7C showed the hematoxylin and eosin (H&E) staining results, and in Ti implant group, the typical features of soft tissue infections included significant acute inflammation and neutrophil infiltration into tissues (marked by yellow arrows) after 808 nm light irradiation for 20 min and after treatment in the dark. However, for the Ti–MoS_2_–IPR group, a relatively milder inflammatory reaction with fewer inflammatory cells was observed after treatment with 808 nm light irradiation. Moreover, the tissue was not damaged, which suggested that the biofilm formed on Ti–MoS_2_–IPR was eradicated successfully and safely using the 808 nm light. In addition, as shown in Figure 7D, the adherent bacteria were observed by Giemsa staining (marked by green arrow). A large number of bacteria were observed in Giemsa‐stained slices regardless of light exposure on the Ti group. By contrast, the amount of bacteria observed in the Ti–MoS_2_–IPR group decreased significantly after the addition of light, verifying the antibacterial ability of Ti–MoS_2_–IPR+Light in vivo.

H&E staining images in Figure S15 (Supporting Information) showed the histological analysis of the major organs after culturing for three days. There were no obvious organ damages or abnormalities, demonstrating the excellent biosafety of the phototherapeutic system.

### 2.6 Osteogenic Activity of the Samples

**Figure 8:**
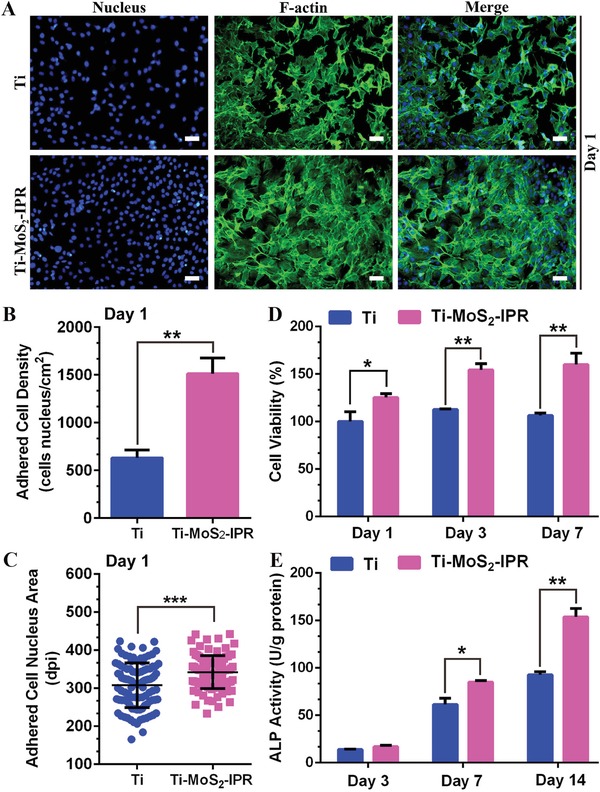
A) Fluorescence images of MC3T3‐El cocultured with different samples for 1 day. Scale bars are 50 µm. B) Analysis of adhered cell density culturing for 1 day, student *t*‐test. C) Analysis of the adhered cell nucleus area culturing for 1 day, student *t*‐test. D) MTT assay for cell proliferation after culture with the samples for 1, 3, and 7 days, two‐way ANOVA with Sidak's multiple comparisons test. E) Quantitative ALP activity of MC3T3‐E1 on different samples after culturing for 3, 7, and 14 days, two‐way ANOVA with Sidak's multiple comparisons test. *p < 0.05, **p < 0.01, and ***p < 0.001.

To investigate the osteogenic activity, MC3T3‐E1 osteoblasts (Tongji Hospital, Wuhan) were seeded on Ti and Ti–MoS_2_–IPR. After being cultured 1 day in the dark, the cells adhered and spread on Ti and Ti–MoS_2_–IPR, with the cell nuclei stained blue and the F‐actin cytoskeleton stained green in **Figure** 8A. After counting the adhered cell nucleus numbers in Figure 8B, the cells were more dense on the Ti–MoS_2_–IPR surface, indicating that the Ti–MoS_2_–IPR surface stimulated the proliferation of the osteoblasts. Previous studies have shown that RGDC is highly effective at promoting the cell adhesion and proliferation, thus accelerating osteogenic differentiation. Actually, cell attachment to a biomaterial is an important early step in the tissue regenerative process. Contacts of cells with neighboring cells and the surrounding microenvironment are mediated by cell adhesion receptors, such as integrin family. RGDC is bioactive ligand and it can bind to multiple integrin species through integrin‐ligand binding affinity to stimulate cell adhesion or mediate cell differentiation significantly.^36^, ^37^ As shown in Figure 8C, the adhered nuclei exhibited a more extended area after the RGDC modification on the implant, which greatly enhanced the osteogenic properties. The cytotoxicity of Ti and Ti–MoS_2_–IPR was assessed using the MTT assay in Figure 8D. Throughout the incubation periods, cells on Ti–MoS_2_–IPR exhibited better cell viability than cells on Ti at days 1, 3, and 7, with the corresponding cell viability reaching ≈160.11% at day 7 relative to the cell viability on pure Ti. This result was attributed to the RGDC modification, which improved the osteoblasts proliferation, and this is also consistent with the cell density results (Figure 8B). Furthermore, the influence of the coating on osteogenic differentiation was analyzed by alkaline phosphatase (ALP) activity. As shown in 8E, cells on Ti–MoS_2_–IPR exhibited higher ALP activity compared with cells on Ti after incubation for 3, 7, and 14 days, and the maximum was observed on day 14, indicating that the introduction of RGDC improved the osteogenic differentiation.

**Figure 9:**
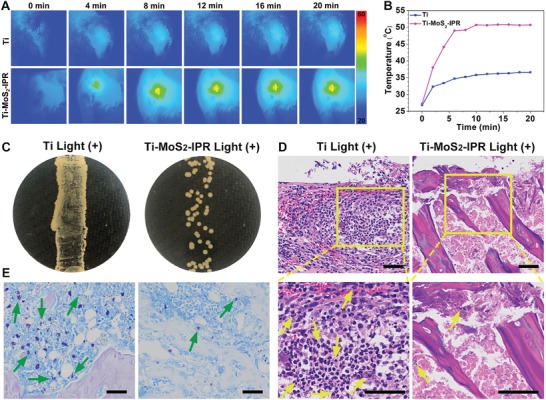
In vivo evaluation of biofilm eradication. A) Infrared thermal images of the Ti and Ti–MoS_2_–IPR groups with light irradiation for 20 min in vivo. B) The corresponding real‐time temperature change in vivo. C) Spread plate results of the Ti+Light and Ti–MoS_2_–IPR+Light rods groups after cultured for three days. D,E) Images of H&E and Giemsa staining infer the degree of infection after treatment with different samples, in which the neutrophils marked by yellow arrows and the bacteria marked by green arrows. Scale bars are 50 µm.

To further investigate the antibiofilm performance and osteogenic properties of the samples in vivo, we utilized *S. aureus* biofilm‐infected rods (Ti and Ti–MoS_2_–IPR) as implants. As shown in Figure S16A (Supporting Information), the rods adhered by biofilm were implanted in the tibia. Computed tomography (CT) image in Figure S16B (Supporting Information) showed the successful implantation of the rods, which were marked by red rectangles. After suturing the wounds, the two groups were irradiated with NIR light for 20 min. **Figure** 9A showed skin temperatures recorded by a thermal imager, which indicated the significant photothermal conversion effect of the Ti–MoS_2_–IPR group in vivo. As shown in Figure 9B, the corresponding temperature in the Ti–MoS_2_–IPR group increased rapidly from 27.3 to 50 °C after 808 nm light irradiation. However, no significant temperature changes were observed in the Ti group. After culturing for three days and then removing the rods and rolling on agar culture plates, the Ti–MoS_2_–IPR group showed several quantifiable bacterial colonies, while the bacterial colonies were spread turbidly in the Ti group (Figure 9C). H&E staining in Figure 9D showed that the Ti implant group included significant acute inflammation and neutrophil infiltration into tissues (marked by yellow arrows) after 808 nm light irradiation. However, for the Ti–MoS_2_–IPR group, fewer inflammatory cells were observed after treatment with light irradiation. Moreover, Giemsa staining in Ti–MoS_2_–IPR group showed fewer adherent bacteria in the tissues (marked by green arrows in Figure 9E). The results indicate that the biofilm on Ti–MoS_2_–IPR sample was substantially eradicated with 808 nm light irradiation in vivo.

**Figure 10:**
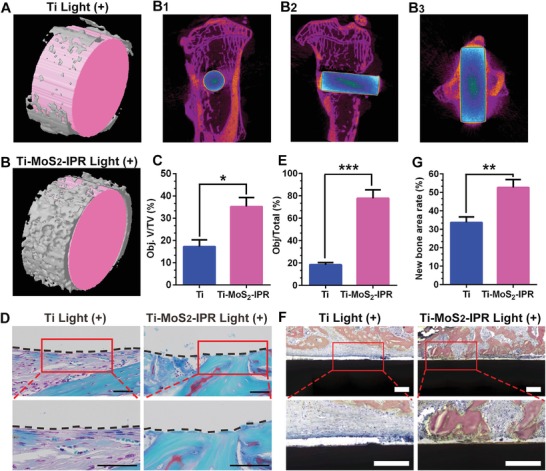
A,B) 3D images reconstructed by micro‐CT in Ti and Ti–MoS_2_–IPR implants. 2D micro‐CT images of Ti–MoS_2_–IPR rods in tibia with the implant being blue (outer) and green (inner), bone being purple: B1) transaxial image, B2) sagittal image, and B3) coronal image closed to the implant surface. C) Quantitative analysis of the newly formed bone volume around the implants, student *t*‐test. D) Safranin‐O/Fast Green staining images show osteogenic ability around Ti and Ti–MoS_2_–IPR implants after 4 weeks implantation. The green color is osteogenesis, and the red or orange color is cartilage (scale bars, 50 µm). E) Quantitative analysis of osteogenesis from histomorphometric measurements, student *t*‐test. F) Histological characteristics at the bone‐implant interfaces stained with methylene blue‐acid fuchsin staining, in which the nucleus of the osteoblast stained into blue and the new bone stained into red (scale bars, 200 µm). G) Quantitative analysis of new bone area rate from the histomorphometric measurements, student *t*‐test. *p < 0.05, **p < 0.01, and ***p < 0.001.

After 4 weeks implantation, microcomputed tomography (micro‐CT) was employed to observe the newly formed bone in **Figure** 10. To decrease the radiation dose and reduce error, three different cylindrical areas (2.5 mm in diameter and 0.9 mm in thickness) around the implant surfaces were used to quantitatively analyze the volume. The reconstructed 3D images showed newly formed bone on Ti (Figure 10A) and Ti–MoS_2_–IPR (Figure 10B) implants with the rod implant being pink (inner) and the newly formed bone being gray (outer). 2D micro‐CT images of Ti–MoS_2_–IPR group depicted the implant in tibia from different directions, as shown in Figure 10B1–B3. The bone network had good continuity with the cancellous bone, and there were newly formed bone tissues detected from bone‐implant interface (being purple in figures), which implies the stimulation of osteogenic activity of the samples. Quantitative analysis significantly suggested that Ti–MoS_2_–IPR group exhibited more stimulation for the formation of new bone tissue (Obj.V (object volume)/TV (tissue volume) = 35.23 ± 4.00%) compared with Ti group (Obj.V/TV = 17.27 ± 2.80%) shown in Figure 10C. Moreover, Safranin‐O/Fast Green staining was used to assess osteogenic differentiation or cartilage differentiation, with the osteogenesis stained by green and cartilage stained by red or orange. As shown in Figure 10D, the Ti–MoS_2_–IPR sample exhibited higher osteogenic differentiation in the bone compared with Ti group. And the quantitative results in Figure 10E indicated that Ti–MoS_2_–IPR sample had the best osteogenesis of about 77.75 ± 6.99%, far higher than the ability of Ti sample (18.45 ± 1.65%). Furthermore, the methylene blue‐acid fuchsin staining performed in Figure 10F was employed to further value the histopathological conditions around the implant, in which the new bone stained into red. The quantitative results showed that the new bone rate in Ti–MoS_2_–IPR group was far higher than the Ti group, with the corresponding value of 33.74 ± 2.86% in pure Ti groups and 52.69 ± 4.12% in Ti–MoS_2_–IPR groups (Figure 10G). Therefore, the Ti–MoS_2_–IPR sample had the largest bone mass compared to pure Ti sample.

## 3 Conclusion

In conclusion, we developed a photoresponsive system combining the biofilm eradication and osteogenic differentiation simultaneously. This hybrid coating system based on magnetron‐sputtered MoS_2_, IR780 photosensitizer, and RGDC exhibits rapid (within 20 min) and effective (98.99 ± 0.42% killing efficiency in vivo) antibiofilm effect under 808 nm light irradiation. Meanwhile, it also exhibited excellent biosafety and osteoconductivity with 77.75% ± 6.99% osteogenesis ratio (according to the quantitative results of Safranin‐O/Fast Green staining), far higher than 18.45% ± 1.65%, the pure Ti sample after 4‐weeks tibia implant. Magnetron‐sputtered MoS_2_ loaded with IR780 can be used for combining PTT and PDT in a single platform without showing noticeable toxicity. The in vitro antibiofilm treatment suggested that oxidative lesions induced by a small quantity of ROS could greatly assist the PTT. In addition, the catalytic activity of GSH oxidation was demonstrated to be accelerated by heat induced by NIR light irradiation, which explains the rapid bacterial death under hyperthermia. Moreover, RGDC modification was also shown to provide the implant with excellent biosafety and osteoconductivity. In conclusion, the combination of multiple antibacterial modalities is considered a promising approach to completely eradicate biofilm through potent synergistic effects without side effects.

## 4 Experimental Section

*Materials*: Medical pure Ti plates (6 mm in diameter, 2 mm in thickness) and rods (2 mm in diameter, 6 mm in thickness) were used as starting substrates. A MoS_2_ target (Φ60 × 5 mm) was purchased from Yan Nuo Xin Cheng, Ltd. (Beijing). Dopamine hydrochloride was acquired from Aladdin Industrial Co. (China). IR780 was obtained from Sigma‐Aldrich. RGDC peptide was purchased from GL Biochem, Ltd. (Shanghai).

*Pretreatment of Ti Surface*: Medical pure Ti plates were first mechanically successively polished and then rinsed several times to remove contaminants. Then, the Ti plates were hydrothermally treated with 4 m KOH at 80 °C oven for 90 min.

*Preparation of the Ti‐MoS_2_ Coating*: MoS_2_ was sputtered on the surface of Ti plates using a JGP‐560a two‐chamber magnetron sputtering system. First, high purity ethanol and acetone were used to clean the Ti substrate several times. When the background vacuum requirement was met, the auxiliary ion source was sputtered on the Ti surface for ≈10 min for further cleaning. Subsequently, under 3.0 Pa sputtering pressure of argon (Ar), the deposition power for MoS_2_ targets was set at 70 W, and the corresponding deposition time was set at 40 min.

*Preparation of the Ti–MoS_2_–IR780–PDA–RGDC Hybrid Coating*: A 20 µL IR780 (0.02 mg mL^−1^) in dichloromethane solution was dropped onto the Ti–MoS_2_ surface, and dried under vacuum. Then the Ti–MoS_2_–IR780 samples were immersed into dopamine hydrochloride solution (2 mg mL^−1^ in 10 × 10^−3^ m Tris‐HCl) and reacted in the dark for 12 h to obtain PDA‐modified Ti–MoS_2_–IR780. After rinsed for several times, samples were dried under vacuum. Subsequently, the samples were placed into RGDC solution (2 mg mL^−1^ in PBS) and reacted in the dark for 12 h. The obtained samples, named Ti–MoS_2_–IR780–PDA–RGDC (Ti–MoS_2_–IPR), were rinsed repeatedly to remove unreacted RGDC, and dried under vacuum.

*Characterization*: FE‐SEM (ZEISS Sigma 500) equipped with EDS and SEM (JSM‐6510LV) were employed to observe the morphology and cross‐sections images. Talosf200 × transmission electron microscope was used to obtain the TEM images. XRD (D8A25, Bruker, Germany) was employed to determine the phase structure of the magnetron‐sputtered samples. Confocal Raman microspectroscopy (Renishaw, UK) was employed to obtain the Raman spectra. XPS (ESCALAB 250Xi, Thermo Scientific, USA) was employed to determine the surface chemical composition. A contact angle instrument (Powereach, JC2000D2) was used to monitor the changes in the surface wettability of the samples after chemical modification at ambient temperature. The UV–Vis–NIR absorbance spectrum was investigated by a UV–Vis–NIR spectrometer (UV–Vis–NIR, UV‐3600, Shimadu, Japan). A light source (808 nm, LOS‐BLD‐0808) was used to induce the photothermal and photodynamic effects. A photothermal imaging measurement of samples was performed using an FLIR E50 instrument (FLIR Systems, Inc., USA). Inverted fluorescence microscope (Olympus, IX73, Japan) was employed to observe fluorescence images.

*Photothermal Effects*: The photothermal effect was measured under continuous 808 nm light irradiation, with the light source at constant power density (0.5 W cm^−2^) and focused to a spot size of 1.6 cm. The Ti, Ti–MoS_2_, Ti–IR780, and Ti–MoS_2_–IPR samples were immersed into 100 µL PBS. During light irradiation, the surface temperature was recorded at 30 s intervals for a total of 10 min using a thermal imager.

*Detection of ROS*: DPBF reacted with ^1^O_2_ to induce a decrease of absorption intensity centered around 410 nm.^58^ Therefore, DPBF was employed to detect the generation of ^1^O_2_ produced by the samples. Briefly, the samples were first immersed in 100 µL DPBF solution (the solvent was dimethyl sulfoxide) for 10 min to reach an adsorption/desorption equilibrium. Then irradiated by NIR light (100 s, 0.5 W cm^−2^), the supernatant of the samples was collected for UV–vis analysis at 25 s intervals.

*Ellman's Assay*: GSH was the major endogenous antioxidant produced by cells, and it was transformed to glutathione disulfide oxidation upon oxidation, preventing cellular damage caused by oxidative stress.^53^, ^54^ Ellman's assay as a versatile method can quantify the thiol groups, and it can be used to determine the possibility of oxidative stress mediated by the samples. Briefly, the samples were immersed in 96‐well plate with 150 µL GSH (0.8 × 10^−3^ m). After reaching the adsorption/desorption equilibrium for 30 min, the samples were irradiated by NIR light (20 min, 0.5 W cm^−2^) or treated in the dark, and the temperature was monitored. H_2_O_2_ (1 × 10^−3^ m) was added to the GSH solution as a positive control. A total of 450 µL of Tris‐HCl (50 × 10^−3^ m, pH 8.0) and 100 µL of 5,5′‐dithio‐bis‐(2‐nitrobenzoic acid) (DTNB) (10 × 10^−3^ m) were added to GSH solution after samples removed. The solution was further blended and even in a rocking bed for 30 min to fully react. The 200 µL of liquid after the reaction was transferred to measure the optical density (OD) at 410 nm. The loss of GSH was calculated according to Equation (1), as follows

(1) $$\mathrm{Loss}\;\mathrm{of}\;\mathrm{GSH}\;\;=\;\;\frac{{\mathrm{OD}}_{\mathrm{negative}\;\mathrm{control}}\;‐\;{\mathrm{OD}}_{\mathrm{sample}}}{{\mathrm{OD}}_{\mathrm{negative}\;\mathrm{control}}}\;\;\times \;\;100\%$$

where GSH solution without samples was defined as the negative control, and OD sample was defined as the absorbance of experimental samples.

*In Vitro Antibiofilm Assay*: A *S. aureus* (ATCC 29 213) biofilm was used to evaluate the antibiofilm characteristics of the samples by the spread plate method. The experimental devices and samples were sterilized with an ultraviolet lamp for at least 30 min. The Ti, Ti–MoS_2_, Ti–MoS_2_–IPR samples were first immersed into 200 µL of bacterial suspension (10^9^ CFU mL^−1^), and subsequently cultured for 48 h at 37 °C. The culture mediums were refreshed every 12 h, and a *S. aureus* biofilm was grown on samples. Then, the samples were immersed in 100 µL of fresh medium with the irradiation of light or without light for 20 min. The nonlight groups were divided into Ti, Ti–MoS_2_, and Ti–MoS_2_–IPR, and the light group were divided into Ti+Light, Ti–MoS_2_+Light, Ti–MoS_2_–IPR+Light (25 °C), and Ti–MoS_2_–IPR+Light (50 °C). Notably, the temperature on Ti–MoS_2_+Light and Ti–MoS_2_–IPR+Light (50 °C) was shown to be five minutes warmer and maintained at 50 °C for 15 min, and the Ti–MoS_2_–IPR+Light (25 °C) group was soaked in ice water bath to maintain a balance of 25 °C between warming and cooling. Then, the bacteria that adhered to the surface of the samples were dispersed in PBS (pH 7.4, 200 µL) via ultrasound. After diluted, 20 µL of bacterial suspensions were spread on LB agar and then placed into a 37 °C incubator for 24 h culturing. By counting the number of colonies (N), Equation (2) was used to calculate the antibacterial ratio as follows

(2) $$\mathrm{Antibacterial}\;\mathrm{ratio}\left(\%\right)\;\;=\;\;\frac{N_{\mathrm{control}}-N_{\mathrm{sample}}}{N_{\mathrm{control}}}\;\;\times \;\;100\%$$

The morphology of the *S. aureus* biofilm was observed by FE‐SEM. Discarding the culture medium after the last step of the antibiofilm assay, the biofilms adhered to the surface of the samples were fixed with 200 µL of glutaraldehyde (2.5%) for 2 h and sequentially dehydrated in alcohol with gradient concentrations. After drying, the samples were sputter‐coated with platinum for FE‐SEM observation. For live/dead staining, the bacteria that adhered to the surface of samples were stained by LIVE/DEAD BacLight bacteria viability kits in the dark for 20 min, washed with PBS, and photographed by fluorescence microscopy.

*Bacterial Membrane Permeability Assay: Ortho*‐nitrophenyl‐β‐galactoside (ONPG) was adopted to observe the change of bacterial membrane permeability on the surface of the samples.^51^, ^52^ After treatment with light or without light irradiation, the samples were analyzed by ONPG assay kit. The supernatant absorbance was measured at 420 nm.

*Cytotoxicity Analysis*: MTT assay was employed to investigate the cytotoxicity of the samples using NIH‐3T3 cells and MC3T3‐E1 osteoprogenitor cells. Before the assay, all samples and experimental devices were sterilized with an ultraviolet lamp about 30 min. On the Ti and Ti–MoS_2_–IPR groups, NIH‐3T3 cells were incubated in Dulbecco's modified Eagle medium/HIGH GLUCOSE (HyClone) medium and cultured at 37 °C incubator for 3 days. MTT solution (0.5 mg mL^−1^, 200 µL) was added to each well after removing the stock solution, and then cultured at cell incubator for 4 h. After sucking out MTT solution, dimethyl sulfoxide (200 µL) was added and then agitated for 15 min. The supernatant was obtained to determine the OD at 490 or 570 nm. The same procedures were also applied to MC3T3‐E1 cells after incubation for 1, 3, and 7 days. Furthermore, to explore the safety of the samples with the NIR light irradiation, a MTT assay using NIH‐3T3 cells was conducted after cells exposed to light for 20 min. The measurements were carried out in triplicate and the cell viability was calculated according to Equation (3) as follows

(3) $$\mathrm{Cell}\;\mathrm{viability}\left(\%\right)\;\;=\;\;\frac{{\mathrm{OD}}_{\mathrm{sample}}}{{\mathrm{OD}}_{\mathrm{control}}}\;\;\times \;\;100\%$$

For morphological observation, NIH‐3T3 cells were incubated with different samples for 3 days. After fixed with 200 µL of glutaraldehyde (2.5%) for 4 h, the cells were dehydrated in alcohol with gradient concentrations (10, 20, 50, 75, 80, 95, and 100%) sequentially. After drying, the samples were observed by FE‐SEM. To examine the adhesion of NIH‐3T3 cells on the substrate, the cells were subjected to immunofluorescent staining analysis. The cells were fixed with formaldehyde (4%) for 20 min and rinsed several times. Then, the fixed cells were subjected to permeabilization in 0.1% (v/v) TritonX‐100 in PBS for 20 min. After rinsed several times, the permeabilized cells were incubated in a bovine serum albumin solution (5% (w/v)) in PBS for 1 h. The samples were immersed in blocking buffer containing primary antibodies against vinculin (1:200 dilution, Proteintech) at 4 °C for 16 h to examine focal adhesions of cells. The cells were then immersed in blocking buffer containing secondary antibodies (1:200 dilution, Proteintech) for 1 h and washed with PBS. Finally, the cytoskeleton was stained by fluorescein isothiocyanate (FITC, YiSen, Shanghai) for 30 min, and the cell nuclei were stained by 4′,6diamidino‐2‐phenylindole (DAPI, YiSen, Shanghai) for 30 s. After rinsing and drying, fluorescence microscopy was employed to photograph the samples. To further detect the cytoskeleton of MC3T3‐E1 cells, the cells were also stained by FITC and DAPI.

*In Vivo Antibiofilm Assay*: Male Wistar rats (180–200 g) were purchased from Hubei Provincial Centers for Disease Prevention & Control, and all procedures were approved by the Department of Orthopedics, Union Hospital, Tongji Medical College, Huazhong University of Science and Technology, Wuhan, China. All animals were treated in accordance with the Animal Management Rules of the Ministry of Health of the People's Republic of China and the Guidelines for the Care and Use of Laboratory Animals of China. The rats were housed in cages for 2 days and randomly divided into two groups as follows: Ti and Ti+Light in one group, Ti–MoS_2_–IPR and Ti–MoS_2_–IPR+Light (50 °C) in other group. After anesthesia with 10% chloral hydrate (4 mL kg^−1^ body weight), the samples coated with already‐formed biofilms were implanted into subcutaneous tissue and then irradiated by 808 nm light for 20 min or treated in the darkness. After stitching up the wound, the rats were fed for 3 days individually. Then, the Ti implants were removed from the subcutaneous tissue and immersed in fresh medium. The bacteria adhered on the surface of implants were dispersed in medium via ultrasound, and 20 µL of bacterial suspensions were spread uniformly on a LB agar and then cultured at incubator for 1 day. Meanwhile, the tissues attached to the implants were harvested using a scalpel and immersed in 4% paraformaldehyde. After dehydration in gradient ethanol solutions and infiltration with xylene, cut tissues were immersed in paraffin wax. Then, the cut tissues in paraffin wax were sectioned by a microtome (Leica RM2016, Leica Microsystems, Germany) to obtain histologic slices. Finally, the histologic sections were stained with H&E and Giemsa stain to assess bacterial contamination of tissues around the implants.

*In Vivo Osteogenic Activity Assay*: A total of 10 rats (400–450 g) were split into Ti+Light and Ti–MoS_2_–IPR+Light groups, and the assay was performed in two legs of the rats. After anesthesia with 10% chloral hydrate (4 mL kg^−1^ body weight), a hole with a diameter of 2.0 mm was drilled into the radius using a hand drill until the marrow was exposure. After swilling the bone cavities with physiological saline, the implants were pressed into the drilled holes. Four rats (two rats from each group) were sacrificed after three days to remove the rods, and the rods were rolled on agar culture plates. After 4 weeks feeding, the others were sacrificed for micro‐CT analysis and histopathological evaluation.

## Conflict of Interest

The authors declare no conflict of interest.

## Supporting information

Supplementary
