# Supplementary material for: Highly Effective and Noninvasive Near‐Infrared Eradication of a Staphylococcus aureus Biofilm on Implants by a Photoresponsive Coating within 20 Min
Source: Adv Sci (Weinh). 2019 Jul 19;6(17):1900599. doi: 10.1002/advs.201900599 (PMC6724470; doi:10.1002/advs.201900599)
Supplement: Supplementary file 1 — Supplementary [file ADVS-6-1900599-s001.pdf]

Copyright WILEY-VCH Verlag GmbH & Co. KGaA, 69469 Weinheim, Germany, 2019.

ADVANCED SCIENCE
Open Access

Supporting Information

for *Adv. Sci.,* DOI: 10.1002/advs.201900599

Highly Effective and Noninvasive Near-Infrared Eradication of a *Staphylococcus aureus* Biofilm on Implants by a Photoresponsive Coating within 20 Min

*Mu Li, Liqian Li, Kun Su, Xiangmei Liu, Tianjin Zhang, Yanqin Liang, Doudou Jing, Xianjin Yang, Dong Zheng, Zhenduo Cui, Zhaoyang Li, Shengli Zhu, Kelvin Wai Kwok Yeung, Yufeng Zheng, Xianbao Wang, and Shuilin Wu**

Copyright WILEY-VCH Verlag GmbH & Co. KGaA, 69469 Weinheim, Germany, 2019.

Supporting Information

Highly Effective and Noninvasive Near-Infrared Eradication of a *Staphylococcus aureus* Biofilm on Implants by a Photoresponsive Coating within 20 Minutes

*Mu Li, Liqian Li, Kun Su, Xiangmei Liu, Tianjin Zhang, Yanqin Liang, Doudou Jing, Xianjin Yang, Dong Zheng, Zhenduo Cui, Zhaoyang Li, Shengli Zhu, Kelvin Wai Kwok Yeung, Yufeng Zheng, Xianbao Wang, Shuilin Wu**

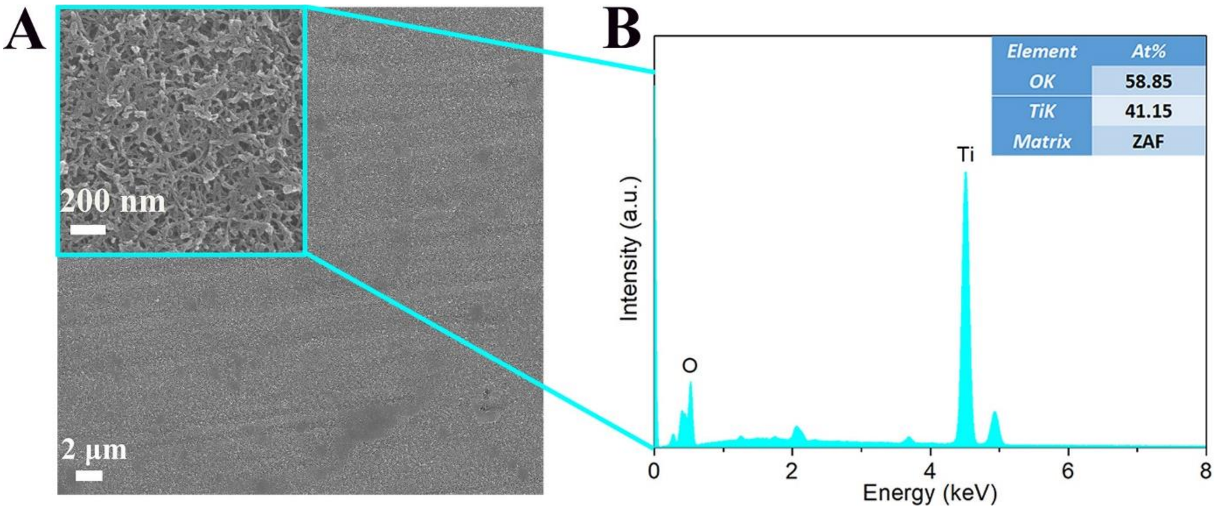

Figure S1. A) FE-SEM images of Ti plates after alkali-heat-treatment, and B) the corresponding EDS image.

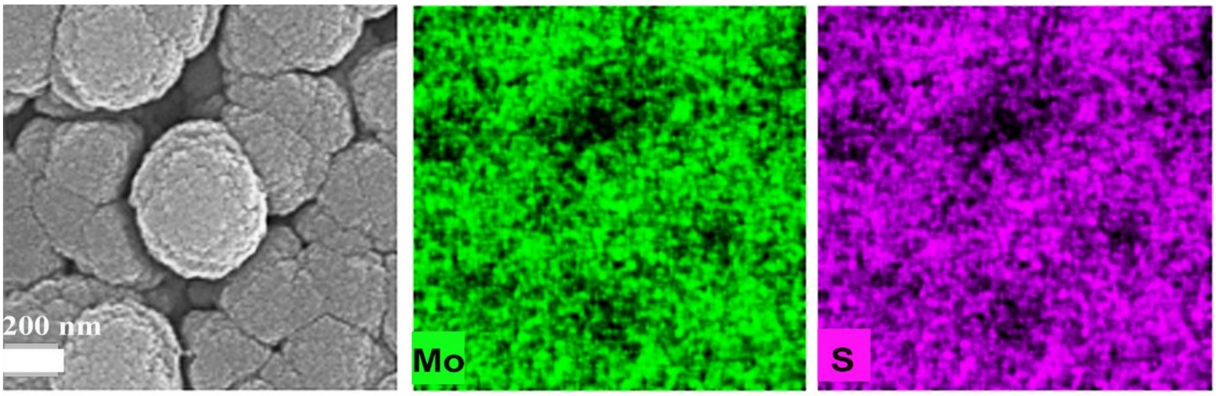

Figure S2. The elemental mapping of Ti-MoS_2 (Mo in green, S in purple) sample.

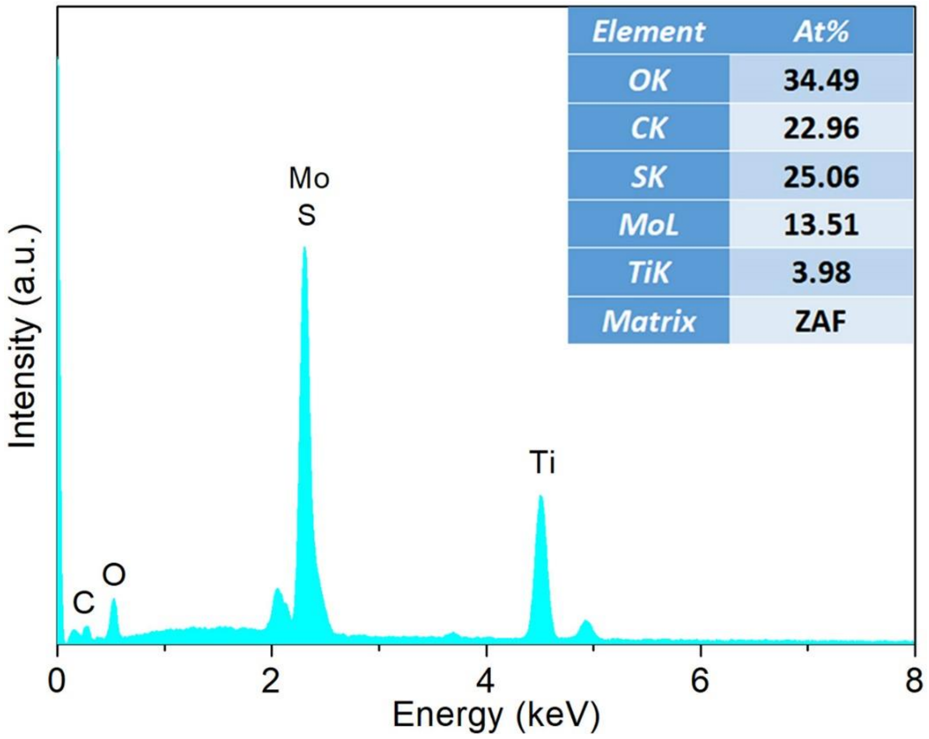

Figure S3. The corresponding EDS image of Ti-MoS_2 sample.

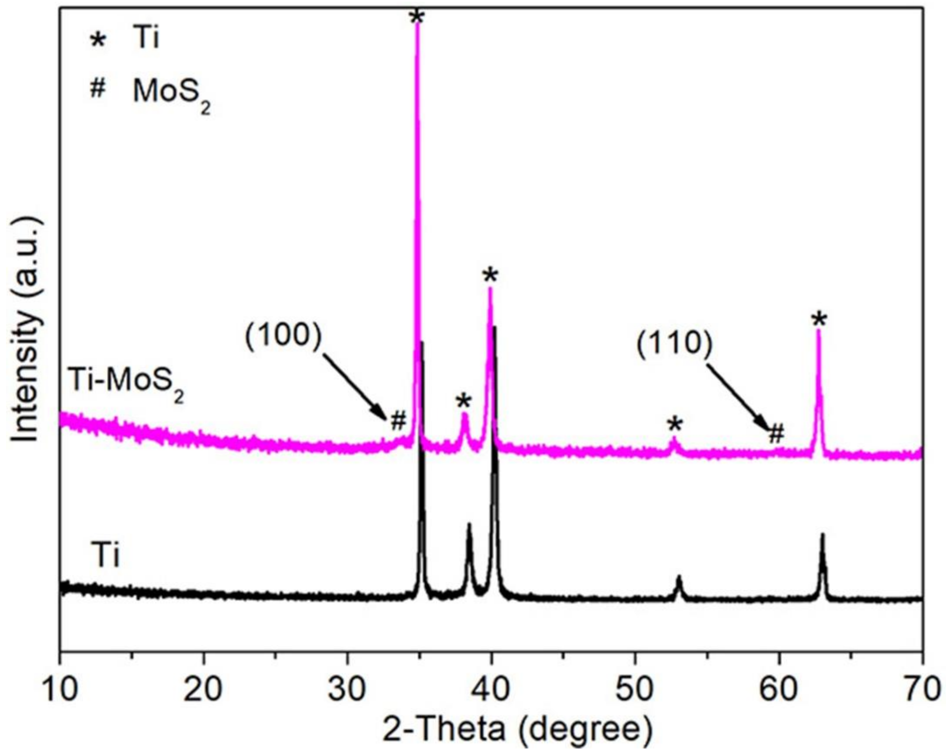

Figure S4. XRD patterns of Ti and Ti-MoS_2 plates.

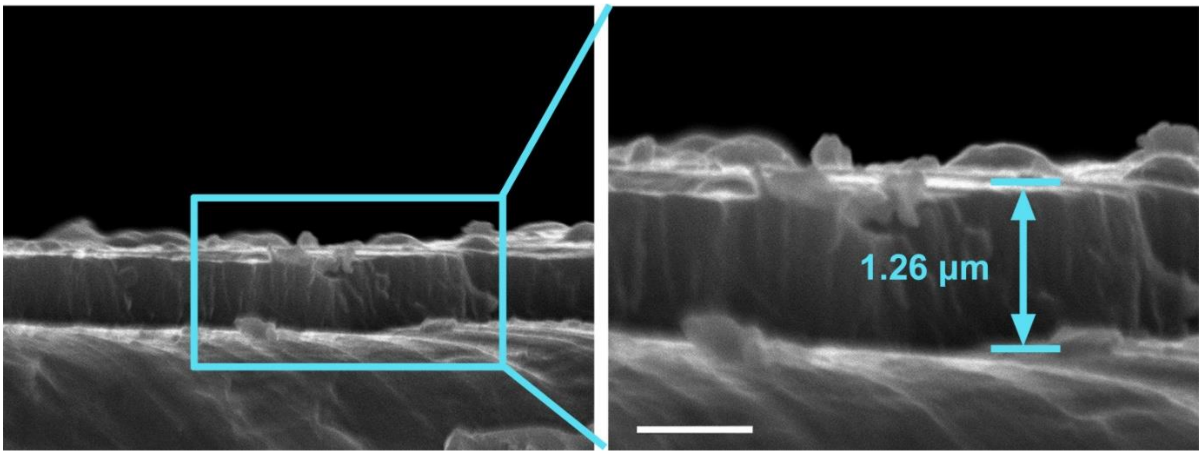

Figure S5. SEM images of the cross-sections in MoS_2 film on Ti plate. Scale bar is 1 μm.

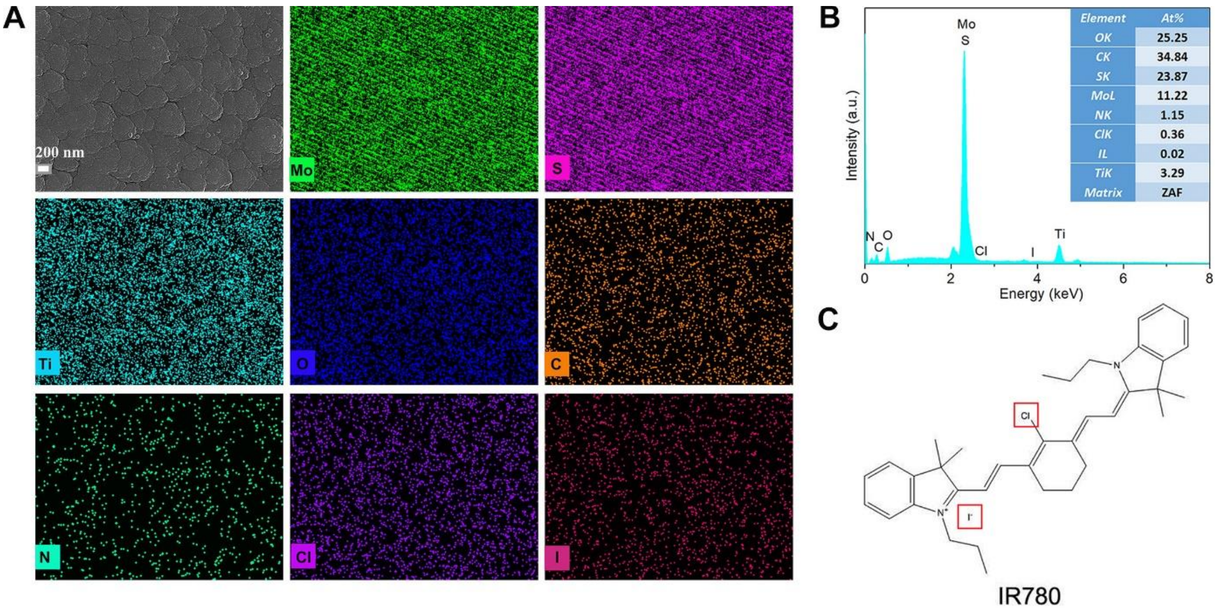

Figure S6. A) The elemental mapping of Ti-MoS_2-IPR, and B) the corresponding EDS image. C) The chemical formula of IR780.

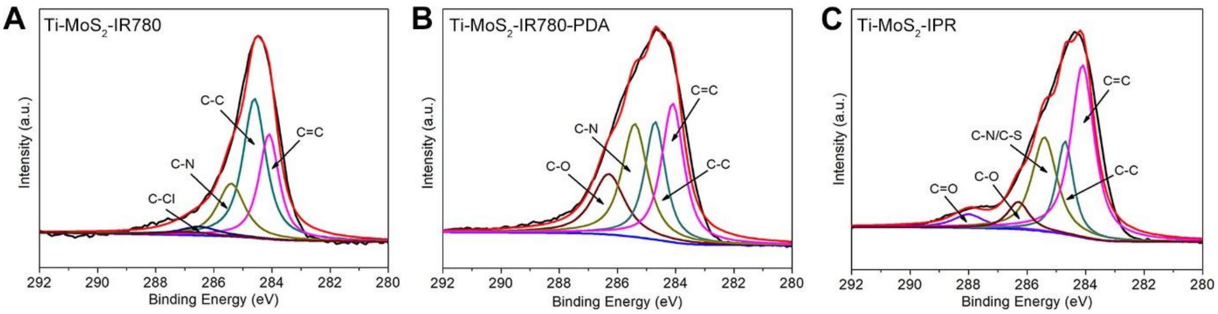

Figure S7. XPS narrow scan spectra of C 1s for A) Ti-MoS_2-IR780, B) Ti-MoS_2-IR780-PDA and C) Ti-MoS_2-IPR.

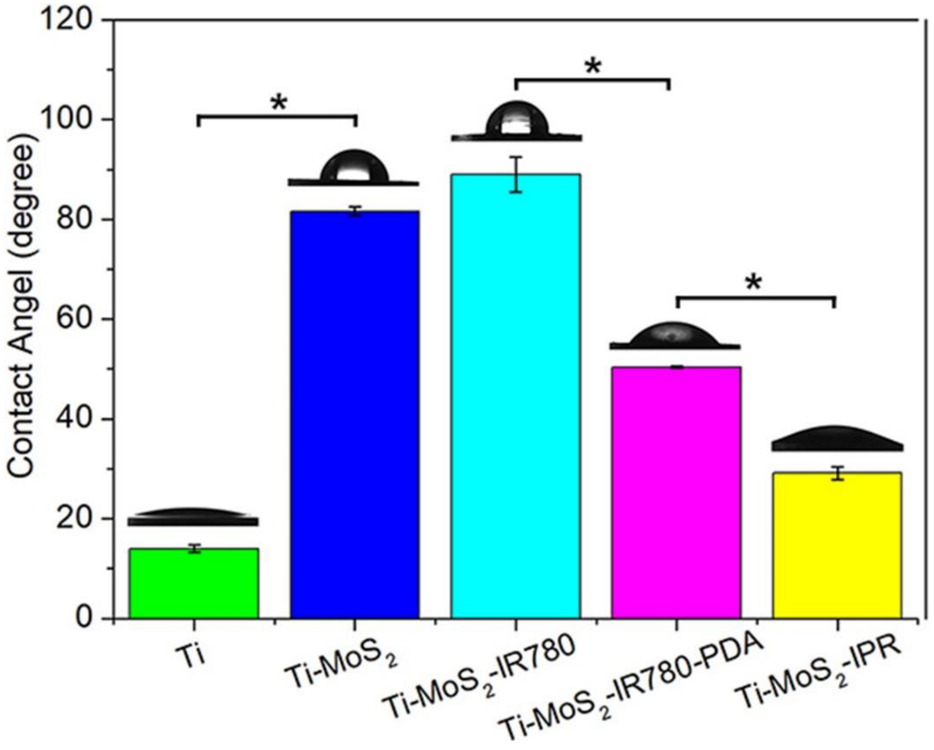

Figure S8. Water contact angle of Ti, Ti-MoS_2, Ti-MoS_2-IR780, Ti-MoS_2-IR780-PDA, and Ti-MoS_2-IPR, student t-test. *$p < 0.05$.

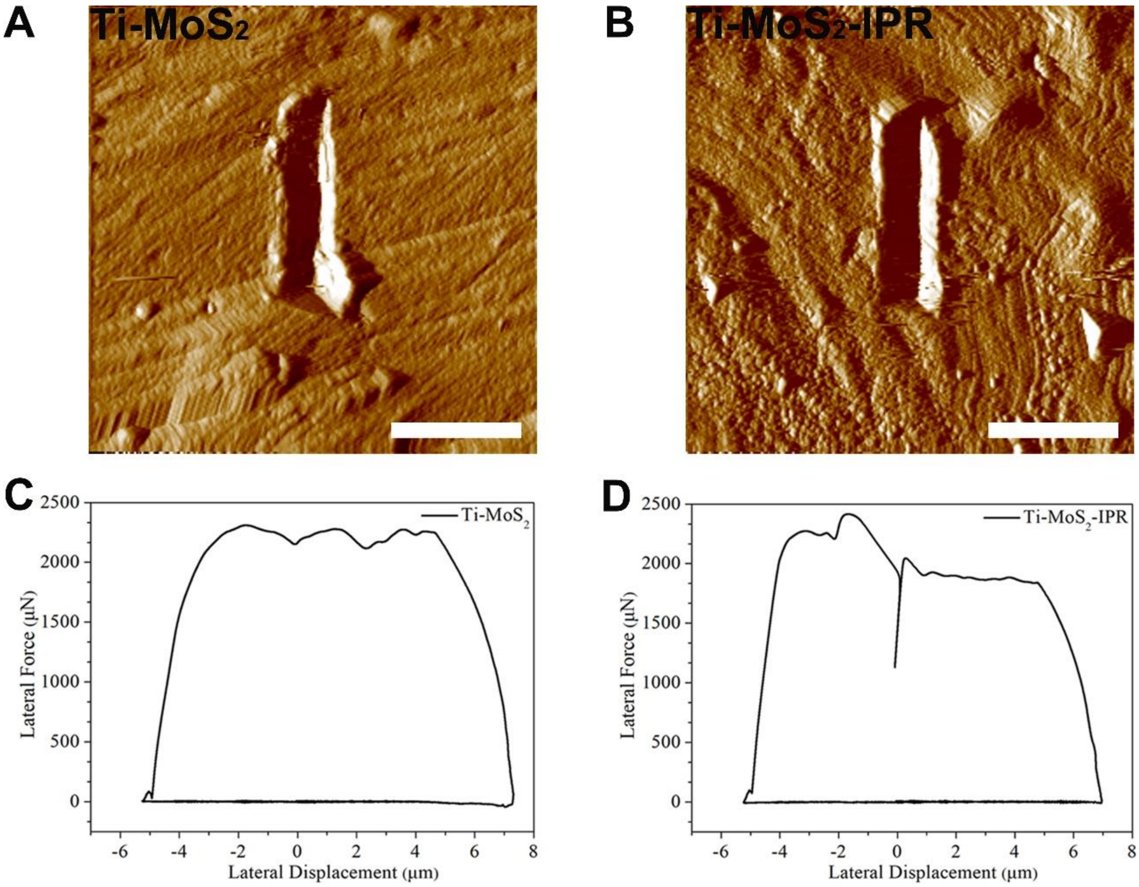

Figure S9. A, B) Morphologies of samples after scratch tested. C, D) Force-displacement curve acquired by the nanoscratch test. Scale bars are 5 μm.

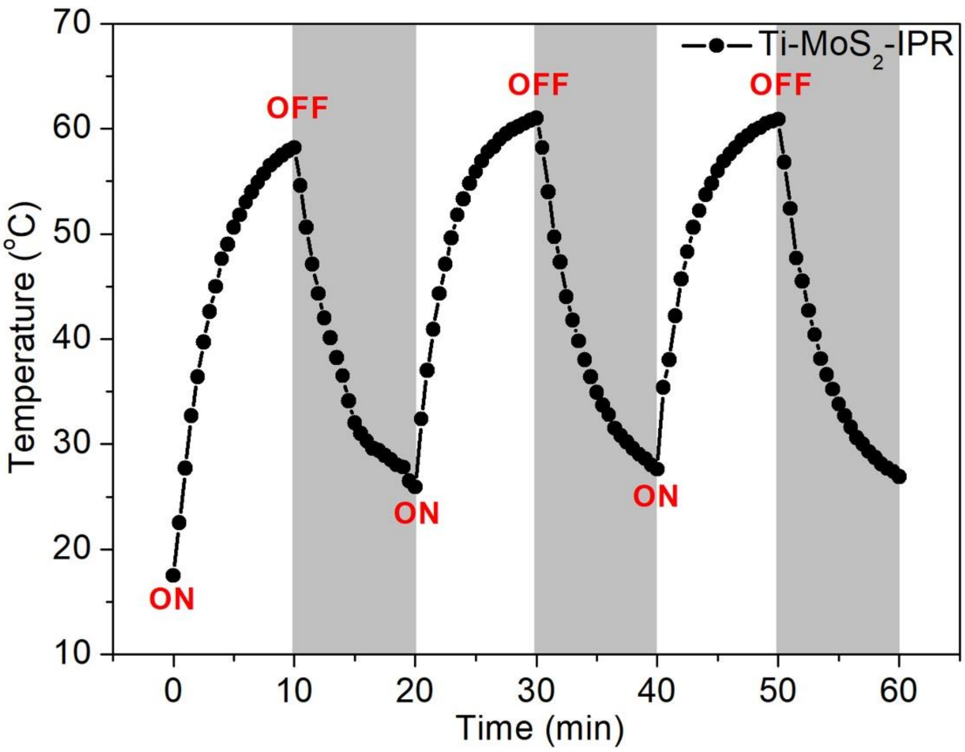

Figure S10. Transient thermal measurements of Ti-MoS_2-IPR under repeated laser on-off cycles of light irradiation (0.5 W cm^{-2}). Three cycles of 808 nm light irradiation were carried out on Ti-MoS_2-IPR and each cycle consists of 10 min irradiation followed by a 10 min cooling phase.

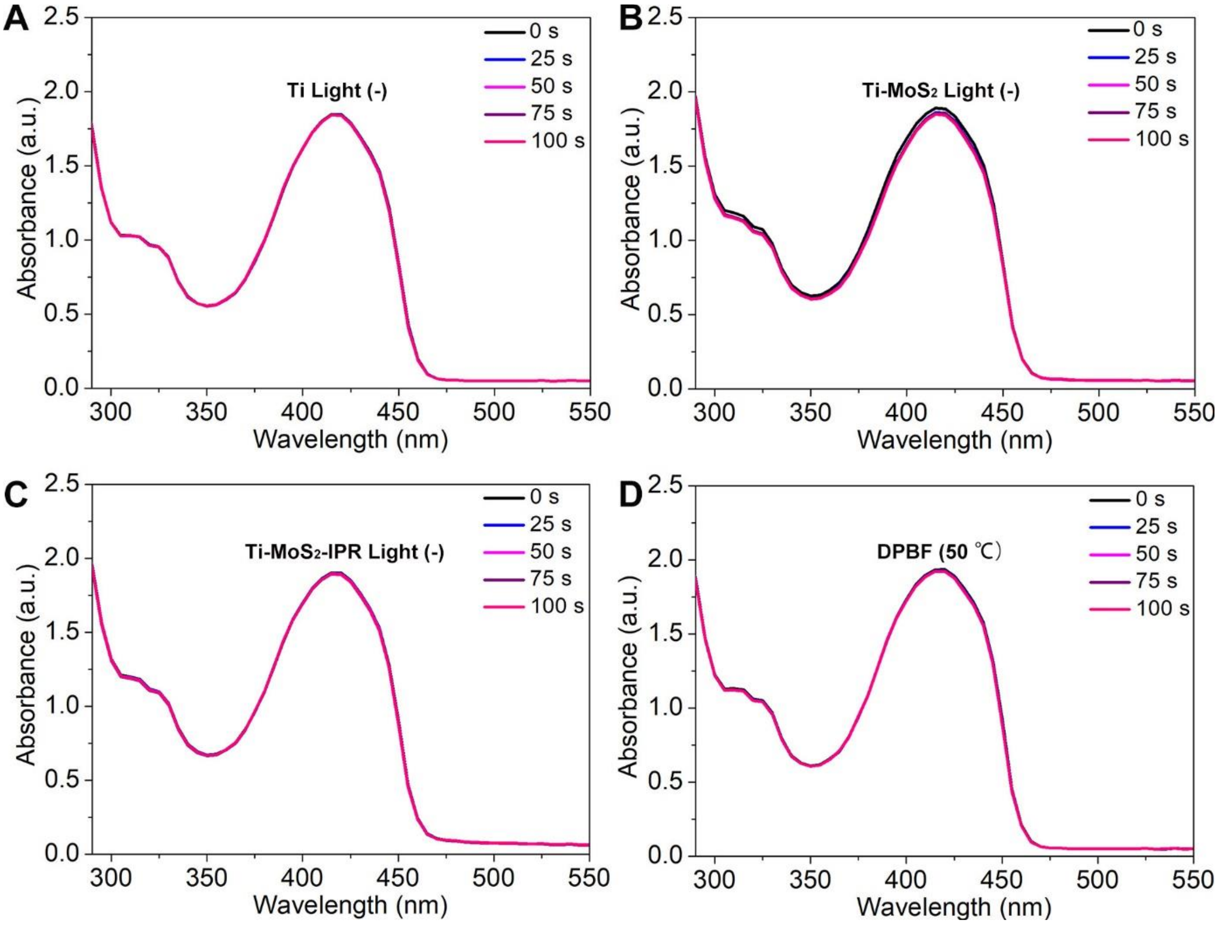

Figure S11. The decay of DPBF for the detection of $^1O_2$ in A) Ti, B) Ti-MoS_2, and C) Ti-MoS_2-IPR in the dark for 100 s. D) Absorbance of the DPBF dye in the absence of samples at 50 °C for 100 s. The experiments were performed in triplicate and independently.

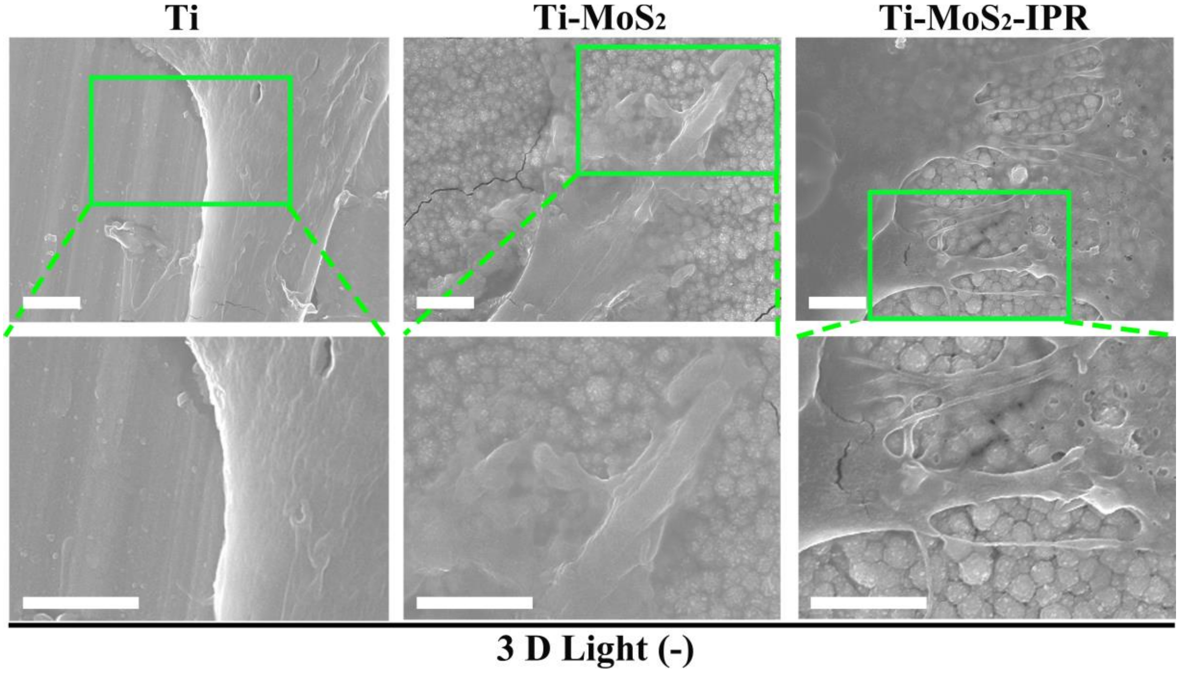

Figure S12. FE-SEM images of cells adhered to Ti, Ti-MoS_2 and Ti-MoS_2-IPR after cultured in the dark for three days. Scale bars are 2 μm.

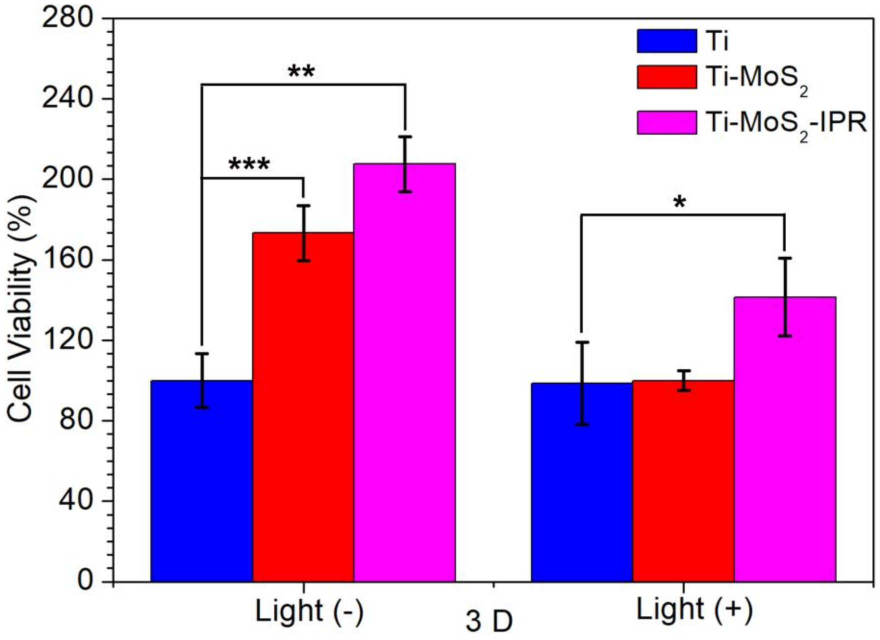

Figure S13. MTT assay of NIH-3T3 cells viabilities on Ti, Ti-MoS_2 and Ti-MoS_2-IPR after treatment in the dark or irradiation by NIR light for 20 min and then cultured for 3 days, two-way ANOVA with Sidak's multiple comparisons test. *$p < 0.05$, **$p < 0.01$.

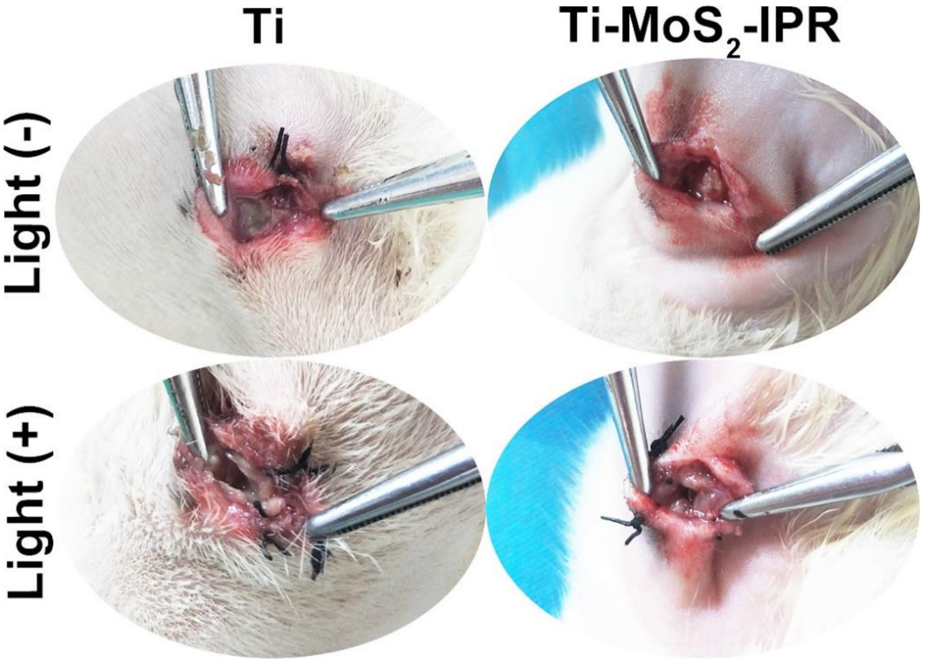

Figure S14. Photographs of the subcutaneous tissue infections after treated with the already-formed biofilm implants of Ti and Ti-MoS_2-IPR at time points of 3 days.

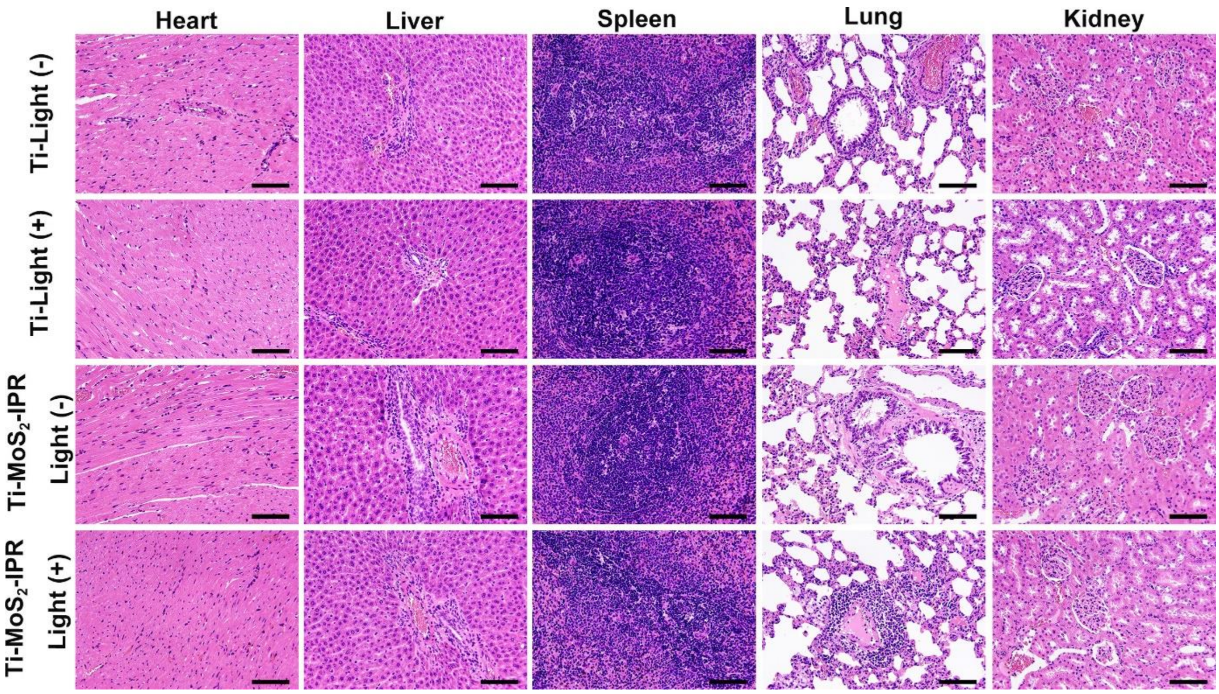

Figure S15. H&E staining images of major organs slices after implantation for 3 days. The experiments are performed in triplicate and independently. (Scale bars, 100 μm).

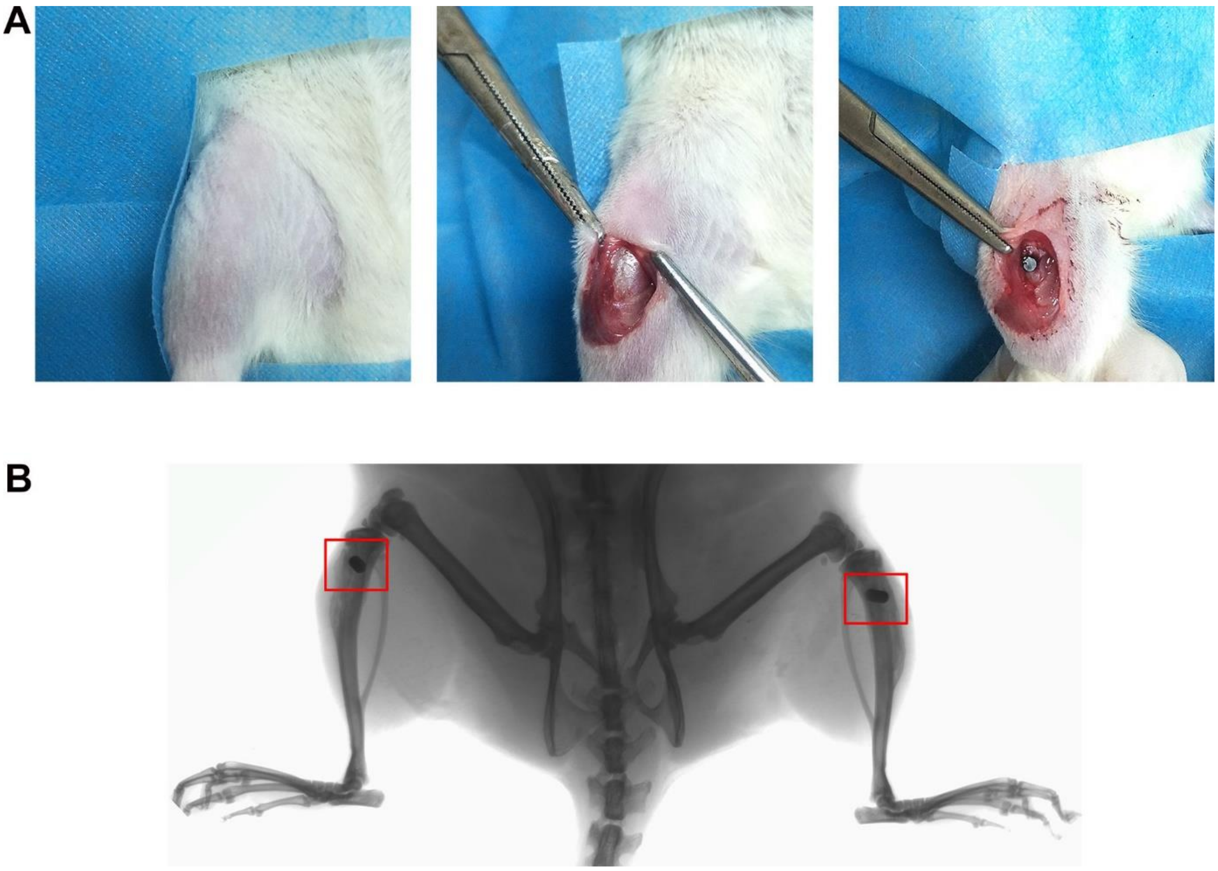

Figure S16. A) Photographs of implantation site *in vivo*. B) CT image of tibia implanted *in vivo*.
